# Considerations when offering mental health first aid to a person with an intellectual disability: a Delphi study

**DOI:** 10.1186/s40359-021-00518-5

**Published:** 2021-02-12

**Authors:** Kathy S. Bond, Fairlie A. Cottrill, Louise Kelly, Joan Broughan, Kimberley Davies, Anna M. Ross, Claire M. Kelly

**Affiliations:** 1 Mental Health First Aid Australia, Parkville, VIC Australia; 2 Centre for Mental Health, Melbourne School of Population and Global Health, University of Melbourne, Parkville, VIC Australia; 3 Flinders University, Adelaide, SA Australia; 4 Victoria University, Melbourne, VIC Australia; 5 Monash University, Melbourne, VIC Australia; 6 Department of Developmental Disability Neuropsychiatry, University of New South Wales, Sydney, Australia

**Keywords:** Mental health problems, Mental illness, Intellectual disability, Mental health first aid

## Abstract

**Background:**

People with an intellectual disability experience higher rates of mental health problems, but experience significant barriers to receiving professional help. Increasing the knowledge and skills of those who support them can help to reduce some of these barriers. This study aimed to develop guidelines for offering mental health first aid to a person with an intellectual disability.

**Methods:**

Using the Delphi research method, a systematic search of websites, books and journal articles was conducted to develop a survey containing items about the knowledge, skills and actions needed for assisting a person with an intellectual disability who is experiencing mental health problems. These items were rated over three survey rounds by an expert panel according to whether they should be included in the guidelines.

**Results:**

Fifty-three experts completed all three survey rounds (67% retention rate). A total of 202 items were rated over the three rounds to yield 170 endorsed items that were incorporated into the guidelines. The developed guidelines emphasise the need to recognise the unique signs of mental health problems in people with an intellectual disability, and provide appropriate support, communication and respect for people with an intellectual disability. The guidelines will also build the capacity of carers to address behaviours of concern, socially limiting behaviours or seeking professional help when the need arises. The guidelines will be used to develop a mental health first aid course.

**Conclusion:**

The guidelines and the resultant mental health first aid course will be a helpful resource with the potential to address some of the barriers to mental health help-seeking that people with an intellectual disability experience.

## Background

Intellectual disability describes a range of neurodevelopmental disorders that impair intellectual and adaptive functioning with onset during childhood [1]. People with an intellectual disability experience higher rates of mental health problems compared to the general population, including higher rates of risk factors for suicide [2, 3]. Australian researchers have found that while disability workers note suicidal behaviour amongst their clients with intellectual disability, few disability services utilise suicide risk assessment tools [5].

Despite a higher prevalence of mental health problems, people with an intellectual disability seek professional help at lower rates than the general public, and even fewer people receive mental health support from services that are specifically for people with an intellectual disability [6, 7]. A number of barriers to accessing mental health services have been identified, including lack of availability of professionals with necessary training and diagnostic overshadowing (symptoms being attributed to the disability rather than to the mental health problem) [8]. Furthermore, symptoms may manifest differently than in people without an intellectual disability [10, 11].

For these reasons, it is important that people who work or live with people with an intellectual disability know how to recognise symptoms of mental health problems (to avoid diagnostic overshadowing and misattributing signs of mental health problems), have the skills to provide appropriate support, and encourage mental health help-seeking. These are the keys skills when providing mental health first aid. Mental health first aid is defined as the help offered to a person developing a mental health problem, experiencing a worsening of an existing mental health problem or in a mental health crisis [12]. The first aid is given until appropriate professional help is received or until the crisis resolves.

Mental Health First Aid (MHFA) Australia has developed training courses that teach mental health first aid skills to the public. People who receive MHFA training have increased mental health knowledge, fewer stigmatising attitudes and show increased supportive behaviours toward individuals with mental health problems [13, 14]. MHFA courses are informed using a series of mental health first aid guidelines developed using the Delphi expert consensus method [15].

The aim of this study was to develop guidelines for how a family member, friend, concerned community member or disability worker without specialist mental health qualifications should give initial assistance (mental health first aid) to a person with an intellectual disability who may be experiencing mental health problems or in a mental health crisis. The guidelines will be used to inform a Mental Health First Aid for Intellectual Disability course.

## Methods

The Delphi expert consensus method was used to develop the guidelines [15]. This particular Delphi methodology has been used to develop a number of similar mental health first aid guidelines [16]. It involved five steps: (1) Literature search (2) Survey development (3) Recruitment of expert panels (4) Data collection and analysis and (5) Guidelines development. The methodology is briefly described below and a more detailed description of the Delphi methodology can be found in the article by Bond and colleagues [17].

### Literature search

A systematic search of grey and academic literature was conducted (including websites, books and academic articles) to collect information about how to offer assistance to someone with an intellectual disability who is experiencing a mental health problem or in a mental health crisis. The grey literature was included because it reflects the wide range of people’s beliefs about intervention and care.

**Fig. 1:**
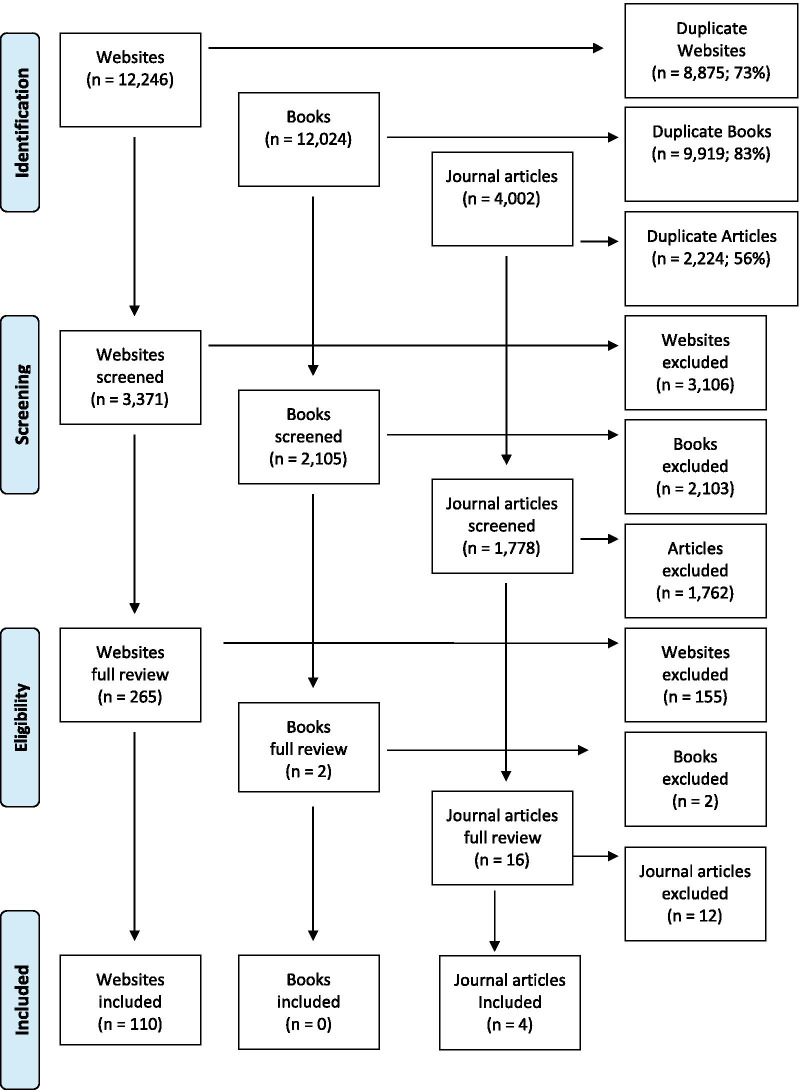
Results of literature search

The search terms were (how to help someone with mental illness OR depression OR anxiety OR psychosis OR suicidal thoughts OR self-injury OR alcohol abuse OR drug abuse OR bipolar OR traumatic event OR eating disorders AND intellectual disability OR developmental disability OR mental retardation OR learning disability) and (first aid for someone with mental illness OR depression OR anxiety OR psychosis OR suicidal thoughts OR self-injury OR alcohol abuse OR drug abuse OR bipolar OR traumatic event OR Eating disorders AND intellectual disability OR developmental disability OR mental retardation OR learning disability). The top 50 websites, books and journal articles for each search was reviewed (see Fig. 1 for results).

### Survey development

The first survey (Round 1) contained statements from the literature search about how a family member, friend, concerned community member or disability worker without specialist mental health qualifications can assist a person with an intellectual disability who may be developing a mental health problem or is in a mental health crisis. The items were drafted by FAC and KSB and then the working group (authors of this paper including Delphi method experts, mental health first aid experts and experts in the field of mental health and intellectual disability) reviewed them to finalise the survey. Survey Monkey was used to administer all surveys (see Additional file 1).

### Panel formation

As per other similar Delphi studies [17] participants were recruited from high-income, western countries that have a Mental Health First Aid program. Participants had to be 18 years or older, be able to read and write in English, and meet the following criteria:

- Have experience in caring (unpaid) for or providing day-to-day support to an adult or adolescent with an intellectual disability who has experienced mental health problems AND have had current or past engagement in activities that give you a broader exposure to the experiences of people with an intellectual disability and mental health problems, e.g. are a member of a carer support group, through a professional role, or carer advocacy organisation.

OR

- Be a mental health professional, disability professional, educator or researcher with at least 3 years’ experience in the area of intellectual disability and mental health.

OR

- Have at least 3 years’ experience working or volunteering for an advocacy organisation in a formal role as an advocate for people with intellectual disability.

A panel of at least 23 has been found to yield stable results [18] and the study aimed to recruit at least 30 to each of the three expert panels (carer, mental health professional or advocate) to allow for attrition. The number of participants recruited to each panel was less than this target. Therefore, all participants were combined into one panel, a reasonable strategy given that the panels’ Round 1 responses were moderately to highly correlated (see Table 1) and the Delphi method aims to recruit panel members who represent a diverse range of relevant expertise [15]. Other similar Delphi studies have employed this strategy as well [17, 19].

**Table 1:** Correlation between experience type

Unpaid carers AND disability advocates	0.66
Unpaid carers AND mental health or disability professional, educator or researcher	0.78
Disability advocates AND mental health or disability professional, educator or researcher	0.84

### Data collection and analysis

Participants rated the aforementioned statements over three rounds using a 5-point Likert scale: ‘Essential’ to be included in the guidelines; ‘Important’ to be included in the guidelines; ‘Don’t know/depends’; ‘Unimportant’; or, ‘Should not be included’ in the guidelines. The specifics of the analysis can be found in the article by Bond and colleagues [17].

### Guidelines development

The first author drafted the guidelines document using the endorsed items and the working group reviewed and finalised the guidelines document. It was given to the expert panel for final approval.

## Results

### Participants

A total number of 79 people were recruited, with 53 completing all three rounds (67.1% retention rate). Participants who completed all three rounds were 60.3% female, 39.6% male and 0% other. They had an average age of 51.2 years (12.36 SD, range 28–84). Participants were from Australia (28.3%), the UK (24.5%), the United States (13.2%), New Zealand (11.3%), Ireland (11.3%), Canada (9.4%) and Sweden (1.9%). Seven (13.2%) participants were MHFA Instructors and the sample had a varied amount of experience with intellectual disability (see Table 2).

**Table 2:** Experience of participants

Experience	Primary role (%)	Secondary role (%)
Unpaid carer	7.6	18.9
Disability advocate	17.0	28.3
Mental health or disability professional, educator or researcher	75.5	5.7
Experienced in more than one role	41.5	

### Item ratings

**Fig. 2:**
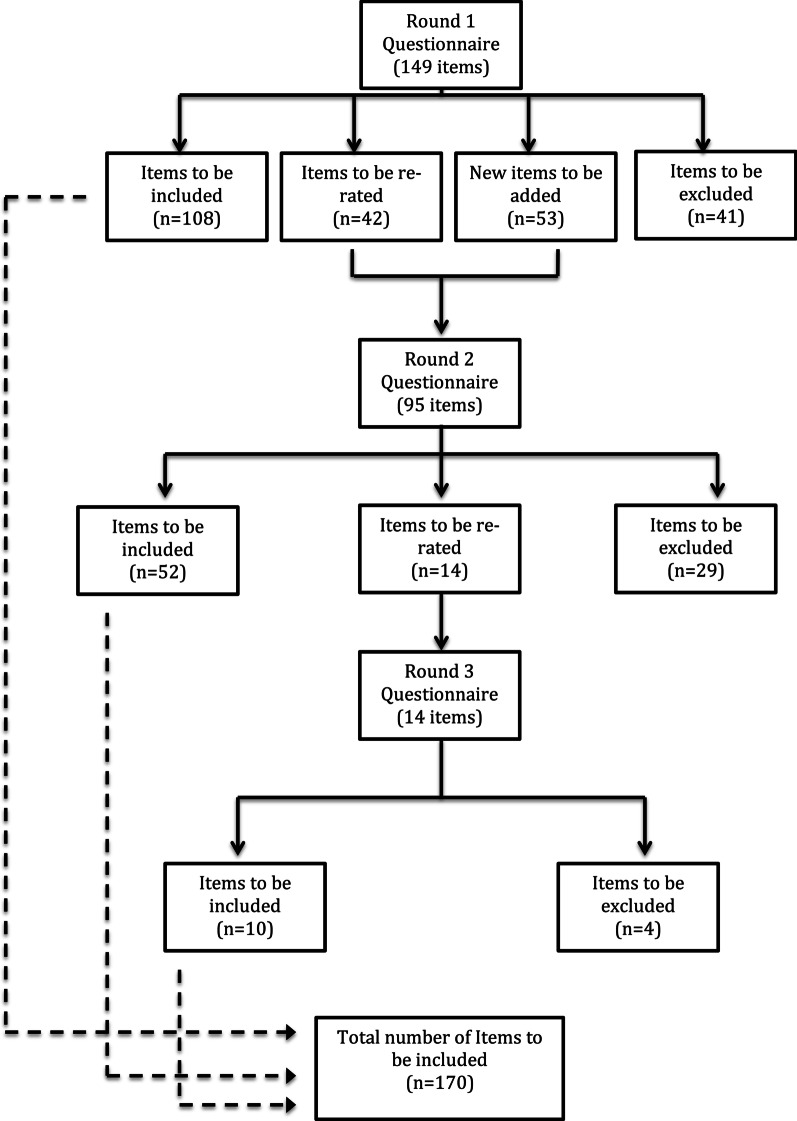
Summary of Item rating

A total of 202 items were rated over the three rounds to yield a total of 170 endorsed items and 74 rejected items (see Additional file 2 for a list of the endorsed and rejected items). Figure 2 presents the information about the total number of items rated, endorsed and rejected over the three rounds. There was a strong positive correlation between the experts in the percentage endorsement for whether items should be included in the guidelines, see Table 1.

### Participant qualitative responses

In Round 1 participants were given the opportunity to provide qualitative feedback that was used to modify or create items to be included in the Round 2 survey. This qualitative feedback highlighted particular areas of concern that needed to be addressed in the guidelines. The complexity of recognising and addressing mental health problems in people with an intellectual disability was highlighted in participant comments, e.g. “Due to complexity of disability and mental health issues, separating and acknowledging both is important.”

Participants felt it was important to emphasise that the person has the same rights as anyone else with a mental health problem. One participant said, “A person with an intellectual disability has the same right to privacy as all others which is only waivered- as for all persons- when harm to self or others is presumed.” As a result of comments like this, the following item was endorsed and included in the guidelines: “The first aider should know that the person's right to privacy should not be waived simply because they have an intellectual disability.”

The participants also highlighted the importance of not making assumptions about the attribution of the person’s behaviour. This was reinforced in the qualitative data as well, e.g. “Changes in behaviour may also be related to physical health issues” and “Also important that changes in behaviour are not assumed to be mental health problems—could be, for example, expressions of pain or discomfort arising from physical health problems.” Thus, the guidelines advise that the first aider “encourage the person to seek medical advice.”

Finally, the guidelines give practical advice for specific situations, something that was highlighted as important by some participants, e.g. “These are important practical steps for a person on the ground working with someone.” For example, advice is given on:

- How to include others (e.g. family members, carers, legal guardians) in a conversation while still maintaining the centrality of the person and their needs.
- Difficulties the first aider may encounter such as aggressive behaviours or sexually inappropriate behaviours.
- What to do if the person is engaging in self-injury, experiencing abuse or trauma, or is suicidal.
- Encouraging appropriate professional help.
- Enhancing communication and comprehension.

### Guidelines development

The endorsed items formed the basis of the guidelines (*Considerations When Offering Mental Health First Aid to a Person with an Intellectual Disability*). The following describes sections and content of the guidelines:

- Knowing about and recognising the signs of mental health problems in people with an intellectual disability.
- Information about specific mental health problems and how they manifest in people with an intellectual disability, including anxiety, hallucinations and delusions, eating disorders, substance use problems and dementia.
- Communicating with the person, including information on enhancing comprehension, engaging others in the conversation, and talking about emotions and mental health problems.
- Supporting the person, including respecting their rights and supporting them to use of self-help strategies.
- Difficulties the first aider may encounter including aggression, sexually inappropriate behaviour and managing personal boundaries.
- What to do if the person is engaging in self-injury, or experiencing abuse or trauma.
- How to encourage professional help.
- What to do in a crisis situation.

There is also specific information for disability workers and what to do if the person needs to be taken to hospital.

The expert panel did not offer any additional comments or suggestions for edits on the guidelines document.

## Discussion

The aim of this study was to develop guidelines for how a family member, friend, concerned community member or disability worker without specialist mental health qualifications should give initial assistance (mental health first aid) to a person with an intellectual disability who may be experiencing mental health problems or in a mental health crisis. These guidelines are being used to form the basis of an MHFA Australia course for members of the public and people who work with people with an intellectual disability, e.g. volunteers, disability carers and advocates. These guidelines and the resultant course will provide clear information on recognising and addressing mental health problems in people with an Intellectual disability.

Providing training on how to recognise and discuss mental health problems with people with an intellectual disability will empower them and those who support them to seek appropriate mental health care. The course may reduce the risk of symptoms being dismissed by workers and carers and diagnostic overshadowing [8] as specific changes and symptoms that indicate a mental health problem can be better recognised and communicated to health professionals.

The guidelines give advice on respecting the rights of the person while engaging with them and their legal guardian. Specifically, the importance of recognising the person’s right to make decisions about their needs (self-determination) is highlighted. Research indicates that self-determination in people with an intellectual disability leads to better quality of life, well-being and improved mental health [20, 21].

These guidelines are to be used in conjunction with other mental health first aid guidelines, including the guidelines for depression [22], suicidal thoughts [23], psychosis [24] and drug and alcohol use problems [25, 26]. In conjunction, the reader is provided with clear, actionable and practical advice on how to support a person with an intellectual disability who is experiencing any number of mental health problems. Furthermore, the course that will be developed using the guidelines will complement the suite of training that is available through Mental Health First Aid Australia, e.g. Mental Health First Aid for young people, older people, people experiencing suicidal thoughts.

## Strengths and limitations

This Delphi study is limited in similar ways to other recent Delphi studies [19, 27], specifically that participants may have been asked to comment on items that were outside their area of expertise and, because there was no opportunity for discussion among participants, biases may remain unchallenged. However, in contrast to this limitation, by eliminating ‘consensus by discussion’, all voices equally influence the endorsement process. This is a major strength of this study.

Another limitation was that it was difficult to recruit enough participants to allow for three separate expert panels, as has been used in other recent Delphi studies [16]. However, it is more important that participants have a diverse range of expertise and, given almost half of participants had experience in two or more of the areas of expertise (professional, unpaid carer, advocate) and given the high inter-panel correlations, this limitation was minimised [15].

## Conclusion

People with an intellectual disability experience mental health problems in similar ways to the general population, however they experience significant barriers to professional help. The mental health first aid guidelines, *Considerations when offering mental health first aid to a person with an intellectual disability,* offer advice on how to recognise when a person with an intellectual disability may be experiencing mental health problems, offer support and encourage professional help. The guidelines were developed using the consensus of 79 experts. They will form the basis of a course that teaches the skills to assist a person with an intellectual disability and mental health problems. It is anticipated that the guidelines and resultant course will be a valuable resource to disability carers, disability workers and members of the public. Future research direction could include evaluation of the course that is based on these guidelines (currently in development) and its impact on course participants and the recipients of mental health first aid.

## Supplementary Information

**Additional file 1**. Copy of R1, R2 and R3 surveys.

**Additional file 2**. Endorsed and Rejected items.

## Data Availability

All data generated or analysed during this study are included in this published article [and its supplementary information files].

## Abbreviations

MHFA: Mental Health First Aid
