# Supplementary material for: Considerations when offering mental health first aid to a person with an intellectual disability: a Delphi study
Source: BMC Psychol. 2021 Feb 12;9:28. doi: 10.1186/s40359-021-00518-5 (PMC7881599; doi:10.1186/s40359-021-00518-5)
Supplement: Supplementary file 1 — Additional file 1. Copy of R1, R2 and R3 surveys. [file 40359_2021_518_MOESM1_ESM.pdf]

Development of guidelines for considerations when providing mental health first aid to a person with an intellectual disability

Information about this research

Purpose of this research

Researchers from Mental Health First Aid Australia and The Centre for Mental Health at the University of Melbourne are collaborating to develop guidelines for how to provide mental health first aid to a person with an intellectual disability. Mental Health First Aid Australia is a not-for-profit organisation focused on mental health training and research. The Centre for Mental Health is based at the Melbourne School of Population and Global Health at the University of Melbourne.

The aim of this research project is to develop mental health first aid guidelines for how a family member, friend, concerned community member or disability worker without specialist mental health qualifications should give initial assistance (mental health first aid) to a person who has an intellectual disability and is experiencing mental health problems.

The guidelines will be available for download on the Mental Health First Aid Australia website (mhfa.com.au) and will be used to inform a specialised Mental Health First Aid course.

How we are doing it

The guidelines will be formed on the basis of expert consensus. We are using the consensus of unpaid carers and significant support people, intellectual disability advocates, and mental health professionals, educators and researchers with expertise in the area of intellectual disability and mental health. These experts will complete online surveys to provide their opinions on a range of strategies for how to help a person with an intellectual disability who may be developing mental health problems or is in a mental health crisis. The strategies that receive a high level of endorsement will be included in the guidelines.

The strategies to be rated in the surveys are obtained from websites, books, fact sheets, brochures, scientific journal articles and training course materials. Some of the statements may seem contradictory or controversial. However, these are included because they reflect the wide range of people's beliefs about intervention and care. They do not necessarily reflect the researchers' opinions.

What will you be asked to do?

If you agree to participate, you will be asked to complete three online surveys over about 4-6 months (so no travel or meetings!) and the total estimated time commitment is approximately 2-3 hours.

Are there any risks?

We do not anticipate that you will experience any risk or discomfort by participating in this research. In the unlikely event that you become distressed at some stage during your participation, you can contact the crisis support service in your country:

Australia: Lifeline on 13 11 14
Canada: National Suicide prevention Lifeline on 1800 273 TALK (8255)
Denmark: Suicide hotline 70 201 201
Finland: SOS Crisis Centre 010 195 202
France: Suicide Écoute 01 45 39 40 00
Germany: TelephoneSeelsorge 0800/111 0 111
The Netherlands: Suicide hotline 0900 0113
New Zealand: Lifeline Aotearoa on 0800 543 354
Republic of Ireland: Samaritans on 116 123
Sweden: Suicide hotline 020 22 00 60
Switzerland: PARSPAS 027 321 21 21
UK: Samaritans on 116 123
USA: National Suicide Prevention Lifeline on 1800 273 TALK (8255)

Do I have to take part?
Participation in this project is voluntary. If you change your mind about participating, you are free to withdraw from the project at any time until the last survey is closed. You may also withdraw your data if you wish, up until the individual survey is closed. Simply contact the project coordinator, Fairlie Cottrill (fairliec@mhfa.com.au).

What will happen to information about me?
Any data we collect from you will be held under password protection and not divulged to others. We are interested in the consensus views of the panels, rather than the views of individual members, so your individual answers will never be reported. We will only present the results in statistical summary form. We occasionally use participant quotes in published journal articles. When this occurs, we do not publish any identifying information with the quote.

Due to research code requirements, we will be storing the information collected for five years after publication, as this is the recommended time period by the University of Melbourne and the Australian Code for the Responsible Conduct of Research. This information will not be deleted, however, until all continued interest in the information (if any at all) ceases.

Who can I contact if I have any concerns about the project?
This research project has been approved by the Human Research Ethics Committee of The University of Melbourne. If you have any concerns or complaints about the conduct of this research project, which you do not wish to discuss with the research team, you should contact the Manager, Human Research Ethics, Research Ethics and Integrity, University of Melbourne, VIC 3010. Tel: +61 3 8344 2073 or Email: HumanEthics-complaints@unimelb.edu.au. All complaints will be treated confidentially. In any correspondence please provide the name of the research team or the name or ethics ID number of the research project.

The ethics ID number of the research project is 1853020.1.

For more information
You received a Plain Language Statement when you expressed interest in this project. Please refer to this for more details about this study.

Development of guidelines for considerations when providing mental health first aid to a person with an intellectual disability

Criteria to participate

Do you meet the criteria to participate in this study?
You have been invited to participate in this research because you are 18 years or over,

AND

- Have experience in caring (unpaid) for or providing day-to-day support to an adult or adolescent with an intellectual disability who has experienced mental health problems AND have had current or past engagement in activities that give you a broader exposure to the experiences of people with an intellectual disability and mental health problems, e.g. are a member of a carer support group, through a professional role, or carer advocacy organisation, etc.

OR

- Are a mental health professional, disability professional, educator or researcher with at least 3 years' experience in the area of intellectual disability and mental health.

OR

- Have at least 3 years' experience working or volunteering for an advocacy organisation in a formal role as an advocate in the area of intellectual disability AND have experience working with people with intellectual disability and mental health problems.

* 1. Do you meet these criteria?

- ◯ Yes
- ◯ No, please exit the survey now.

Development of guidelines for considerations when providing mental health first aid to a person with an intellectual disability

Consent

Consent to participate
It is important for you to know that participation in this study is voluntary. You are not under any obligation to participate and you can withdraw at any time until the last survey is closed.

Best wishes,
The Mental Health First Aid Research Team

1. I consent to participate in this project, the details of which have been explained to me, and I have been provided with a written plain language statement to keep.

2. I understand that the purpose of this research is to develop Mental Health First Aid Guidelines for providing mental health first aid to a person with an intellectual disability.

3. I understand that my participation in this project is for research purposes only.

4. I acknowledge that the possible effects of participating in this research project have been explained to my satisfaction.

5. In this project I will be required to complete three online surveys over about 4-6 months and the total estimated time commitment is approximately 2-3 hours.

6. I understand that my participation is voluntary and that I am free to withdraw from this project anytime without explanation or prejudice and to withdraw any unprocessed data that I have provided.

7. I understand that the data from this research will be stored at Mental Health First Aid Australia and will be destroyed after 5 years.

8. I have been informed that the confidentiality of the information I provide will be safeguarded subject to any legal requirements; my data will be password protected and accessible only by the named researchers.

9. I understand that given the small number of participants involved in the study, it may not be possible to guarantee my anonymity, but no personal or identifying data will be published or accessible by anyone outside the research team.

10. I understand that after I consent to participating, any information I provide will be retained by the researcher for five years after the publication of the project.

* 2. I understand that by submitting this survey I am giving my consent to participate in this study.

- ◯ Yes, I understand.
- ◯ I do not consent to participating in this research. Please exit the survey now.

Development of guidelines for considerations when providing mental

Instructions

How this questionnaire was developed

The statements in this questionnaire were derived from information collected during a literature search of websites, books, and journal articles on how to provide mental health first aid to somebody who has an intellectual disability and is experiencing mental health problems. This search examined any written information on this topic.

Some of the statements may seem contradictory or controversial; however, they have included them because they reflect the wide range of people's beliefs about the best ways to provide mental health first aid to a person who has an intellectual disability and is experiencing mental health problems. It is important to note that the researchers have not made judgements about the statements in the survey and do not necessarily agree or disagree with them. The purpose of the survey is to obtain a consensus view about each of the statements from the expert panel. You have been invited to be a member of the expert panel to inform the development of a set of guidelines that reflect current expert opinion.

You will note that there is a place for you to add comments at the end of each section. This is so you can suggest any additional helping statements you think are important to providing mental health first aid to someone who has an intellectual disability and is experiencing mental health problems. These statements will then go into a second survey to be rated by the expert panels.

Definitions used in this survey

Intellectual disability: is characterised by:
Global deficiencies in intellectual and adaptive functioning, where the person may not reach expected developmental milestones. The deficiencies in the intellectual functions and adaptability begin during childhood or adolescence.

Mental health first aid: is the help offered to a person developing a mental health problem, experiencing a worsening of an existing mental health problem, or in a mental health crisis. The first aid is given until appropriate professional help is received or until the crisis resolves.

The person: a person who has an intellectual disability, who the mental health first aider (first aider) is concerned may be experiencing a mental health problem.

The first aider: a family member, friend, concerned community member or disability worker without specialist mental health qualifications who provides initial help to a person who has an intellectual disability and is experiencing mental health problems.

Carer or support person: refers to people who provide paid or unpaid personal care, support and assistance to a person with an intellectual disability. It can include a spouse, de facto partner, parent, other relative or guardian, or disability support worker.

Disability worker: a person paid to support someone with an intellectual disability. They may or may not be formally trained.

GP/Family doctor: a medical doctor based in the community who treats patients with minor or chronic illnesses and refers those with serious conditions to a specialist or hospital.

Professional/health professional: a broad range of health professionals through which a person may seek help for mental health problems. This could include a mental health professional, GP/family doctor, or another health professional, e.g. allied health professional, hospital emergency staff.

Mental health professional: a health professional who is qualified to treat people who are experiencing mental health problems, e.g. a psychologist, mental health nurse, psychiatrist, or social worker or occupational therapist with specialist mental health training.

Emergency services: services that respond to and deal with emergencies when they occur, e.g. emergency medical services (ambulance) or law enforcement (the police).

Mental health crisis service: services that respond to and provide immediate help during a mental health crisis and are responsible for assessing the care required by the person. Psychiatric nurses, social workers, psychiatrists and psychologists may work for a mental health crisis service.

Mental health crisis/crisis: a situation in which:
- The person may harm themselves, e.g. by attempting suicide, by using substances to become intoxicated, by engaging in non-suicidal self-injury, or as a result of extreme weight loss
- The person experiences extreme distress, e.g. a panic attack, a traumatic event or a severe psychotic state
- The person's behaviour is very disturbing to others, e.g. they become aggressive or lose touch with reality.

Abuse: mistreatment that occurs between people (interpersonal trauma), e.g. emotional, physical or sexual abuse including family violence, child abuse, and elder abuse.

Trauma: an emotional response to a powerful and distressing experience. Immediate trauma responses can include shock and denial. Longer term reactions include unpredictable emotions, flashbacks, strained relationships and physical symptoms like headaches or nausea. Powerful and distressing experiences are usually life threatening or pose a significant threat to a person's physical or psychological wellbeing.

Some common examples events that have the potential to cause trauma include interpersonal violence (including family violence, child abuse, elder abuse, physical or sexual assault, mugging or robbery), accidents (such as traffic or workplace accidents), and witnessing something terrible happen. Mass traumatic events include war, terrorist attacks, mass shootings, and severe weather events (flood, earthquake, hurricane, tsunami, forest and bush fire).

Indirect exposure can also cause trauma, for example witnessing others experience a potentially traumatic event, learning that a potentially traumatic event occurred to someone you know, or repeated or extreme exposure to details of a potentially traumatic event.

Non-suicidal self-injury: self injury that is not intended to result in death. It does not include behaviours that may cause unintentional injury to the person (e.g. stimming behaviours).

Self-injury/self-injurious behaviour: behaviour that has the potential to cause physical injury to oneself and can be for a range of reasons, including to communicate a need such as hunger, to manage distress (NSSI), or as a result of stimming behaviours.

Self-stimulatory behaviour (stimming): the repetition of physical movements, sounds, or repetitive movement of objects common in people with an intellectual disability. Stimming behaviours include hand flapping, rocking, excessive or hard blinking, pacing, head banging, hitting the head, pulling the hair, repeating noises or words, snapping fingers, and spinning objects. While stimming can be an outlet for the person - e.g. to gain/reduce sensory input if the person has sensory sensitivities, some stimming behaviours can cause unintentional injury to the person (e.g. hitting the head or pulling the hair).

'*Challenging behaviour*': Behaviour of such intensity, frequency or duration that the physical safety of the person or others is placed in serious jeopardy, or behaviour which is likely to seriously limit or deny access to the use of ordinary community facilities. It can include a range of behaviours, for example physical aggression towards objects or people, self-injury, sexually inappropriate behaviour, offending behaviour (such as arson or stealing), mannerisms or ritual.

Development of guidelines for considerations when providing mental health first aid to a person with an intellectual disability

Instructions

Instructions

There are already guidelines to help people who may be experiencing a range of mental health problems (go to our website to access these guidelines). We do not wish to replicate existing MHFA guidelines. Rather, the aim of this project is to develop guidelines on how to tailor mental health first aid to help a person who has an intellectual disability and is experiencing mental health problems. The guidelines that results from this project will be used in conjunction with the existing mental health first aid guidelines.

Please complete the questionnaire by rating each statement according to how important you believe it is for inclusion in the guidelines for considerations when providing mental health first aid to a who has an intellectual disability and is experiencing mental health problems. Please keep in mind that the guidelines will be used by family members, friends, concerned community members or disability workers without specialist mental health qualifications. The guidelines will not be for mental health professionals who are looking for guidance on working with patients with intellectual disabilities.

The statements need to be rated according to their importance for someone ***WITHOUT a counselling or clinical background.***

This questionnaire should take approximately 1-2 hours to complete. You can complete the survey in two or more sittings. Your answers are saved when you click 'Next' at the bottom of a page. This marks your page and you can begin again at a later date on the next page. **Please be aware that once you have logged on and started responding you must complete the questionnaire on the same computer.**

In the next phase of the research you will be asked to complete another two surveys over approximately 6 months. The following two surveys will be considerably shorter and take less time to complete.

Overview of the questionnaire

Section 1: Knowing about intellectual disability and mental health problems
Section 2: Recognising and acknowledging a person may be experiencing mental health problems
Section 3: Raising concerns with the person
Section 4: Communicating with the person
Section 5: Respect and rights
Section 6: Being supportive
Section 7: Psychosis
Section 8: Anxiety
Section 9: Self-injury
Section 10: Substance use
Section 11: Eating disorders
Section 12: Dementia
Section 13: Abuse
Section 14: Trauma
Section 15: Other supports
Section 16: Difficulties the first aider may encounter
Section 17: Professional help
Section 18: Crisis situation
Section 19: If the person needs to be taken to hospital

Development of guidelines for considerations when providing mental health first aid to a person with an intellectual disability

Information about you

* 3. What is your name? (This allows us to determine who has completed the Round 1 survey and is therefore eligible to participate in Round 2. Your name will be deleted from your data when the project is complete).

* 4. How old are you?

* 5. What is your gender?

- Female
- Male
- I identify with another term
- Do not wish to disclose.

* 6. Please indicate your primary source of expertise, e.g. Unpaid carer or significant support person, Disability advocate or Intellectual disability mental health professional.

- Unpaid carer or significant support person for someone who has an intellectual disability and has experienced mental health problems.
- Disability advocate with experience working with people with intellectual disability and mental health problems
- Mental health professional, disability professional, educator or researcher in the area of intellectual disability and mental health.

* 7. In addition to your primary source of expertise, do you also have experience in intellectual disability <u>and</u> mental health as a: (tick all that apply)?

- [ ] Unpaid carer or significant support person for someone who has an intellectual disability and has experienced mental health problems
- [ ] Disability advocate with experience working with people with intellectual disability and mental health problems
- [ ] Mental health professional, disability professional, educator or researcher in the area of intellectual disability and mental health

* 8. Please state the name of the organisation/s you work or volunteer for that make you eligible to participate in this study?

* 9. What is your role within the above organisation/s?

* 10. What country do you live in?

* 11. Are you a Mental Health First Aid Instructor, i.e. do you deliver the Mental Health First Aid course?

- Yes
- No

KNOWING ABOUT INTELLECTUAL DISABILITY AND MENTAL HEALTH PROBLEMS

This section contains statements about what the first aider needs to know about intellectual disability and mental health problems

Please rate how important (from ‘essential’ to ‘should not be included’) you think it is that each statement be included in the guidelines.

Please keep our definitions in mind when responding to this section. You can access the definitions here.

Knowing about intellectual disability and mental health problems

* 12. The first aider should know that people with an intellectual disability are more likely to experience mental health problems than the general population.

- Essential
- Important
- Don't know/depends
- Unimportant
- Should not be included

* 13. The first aider should know that the likelihood of developing a mental health problem is higher in people with more severe intellectual disability.

- Essential
- Important
- Don't know/depends
- Unimportant
- Should not be included

14. Please provide any additional items or comments related to this section.

Development of guidelines for considerations when providing mental health first aid to a person with an intellectual disability

RECOGNISING AND ACKNOWLEDGING A PERSON MAY BE EXPERIENCING MENTAL HEALTH PROBLEMS

This section contains statements about what the first aider needs to know about recognising and acknowledging that a person with an intellectual disability may be experiencing mental health problems.

Please rate how important (from ‘essential’ to ‘should not be included’) you think it is that each statement be included in the guidelines.

Please keep our definitions in mind when responding to this section. You can access the definitions here.

Recognising and acknowledging a person with an intellectual disability may be experiencing mental health problems.

* 15. The first aider should know the signs and symptoms of mental illness in people with an intellectual disability.

○ Essential
○ Important
○ Don't know/depends
○ Unimportant
○ Should not be included

* 16. If the first aider notices changes in the person's behaviour they should consider a mental health problem as a possible reason, and not dismiss these changes as part of the intellectual disability.

○ Essential
○ Important
○ Don't know/depends
○ Unimportant
○ Should not be included

* 17. The first aider should not dismiss changes in the person's behaviour as part of their intellectual disability.

○ Essential
○ Important
○ Don't know/depends
○ Unimportant
○ Should not be included

* 18. The first aider should not assume that any one change in the person indicates a mental health problem, rather consider the frequency and severity of the changes.

- Essential
- Important
- Don't know/depends
- Unimportant
- Should not be included

* 19. The first aider should know that people with an intellectual disability experience mental health problems in the same way that other people do, however the symptoms may present differently.

- Essential
- Important
- Don't know/depends
- Unimportant
- Should not be included

* 20. The first aider should know that seemingly minor changes or incidents can have a significant impact on a person with an intellectual disability.

- Essential
- Important
- Don't know/depends
- Unimportant
- Should not be included

* 21. If the first aider does not know the person well and is not sure if the signs or symptoms they have noticed are unusual for that person, they should consult others who provide support (e.g. colleagues or the person’s guardian or family), with the person's permission.

- Essential
- Important
- Don't know/depends
- Unimportant
- Should not be included

22. Please provide any additional items or comments related to this section.

PLEASE NOTE:

The following three statements apply **only** to situations in which the first aider is a **paid disability worker.**

Please rate how important (from 'essential' to 'should not be included') you think it is that each statement be included in the guidelines when the first aider is a **paid disability worker.**

* 23. If the first aider needs to record information about any changes in the person's behaviour or mood, they should do this in a factual way rather than giving an opinion, and only with the person's permission.

- Essential
- Important
- Don't know/depends
- Unimportant
- Should not be included

* 24. The first aider should talk to colleagues to see if anyone else has noticed similar changes in the person's behaviour or mood.

- Essential
- Important
- Don't know/depends
- Unimportant
- Should not be included

* 25. The first aider should discuss the changes they have noticed in the person with a case manager or employer.

- Essential
- Important
- Don't know/depends
- Unimportant
- Should not be included

26. Please provide any additional items or comments related to this section.

RAISING CONCERNS WITH THE PERSON

This section contains statements about what the first aider needs to know about raising their concerns with the person.

Please rate how important (from ‘essential’ to ‘should not be included’) you think it is that each statement be included in the guidelines.

Please keep our definitions in mind when responding to this section. You can access the definitions here.

Raising concerns with the person

* 27. If the first aider offers to help the person and the person does not want their help, the first aider should respect this.

- Essential
- Important
- Don't know/depends
- Unimportant
- Should not be included

* 28. The first aider should be aware that the person may have a limited understanding of mental health problems.

- Essential
- Important
- Don't know/depends
- Unimportant
- Should not be included

* 29. The first aider should be aware that the person's family, carers or other support workers may have limited understanding of mental health problems.

- Essential
- Important
- Don't know/depends
- Unimportant
- Should not be included

* 30. The first aider should not assume they know the best way of helping the person and should be guided by any instructions the person or their legal guardian may give.

- Essential
- Important
- Don't know/depends
- Unimportant
- Should not be included

31. Please provide any additional items or comments related to this section.

Development of guidelines for considerations when providing mental health first aid to a person with an intellectual disability

COMMUNICATING WITH THE PERSON

This section contains statements about what the first aider needs to know and do when communicating with the person about their symptoms.

Please rate how important (from 'essential' to 'should not be included') you think it is that each statement be included in the guidelines.

Please keep our definitions in mind when responding to this section. You can access the definitions here.

There are four parts to this section:
1. Communicating with the person
2. Comprehension
3. Questions
4. Engaging others in the conversation

Communicating with the person

* 32. The first aider should know how intellectual disability may impact communication and comprehension.

- Essential
- Important
- Don't know/depends
- Unimportant
- Should not be included

* 33. The first aider should try to find out as much as possible about the person's communication style and preferences.

- Essential
- Important
- Don't know/depends
- Unimportant
- Should not be included

* 34. The first aider should not make assumptions about the person's communication style and preferences.

- Essential
- Important
- Don't know/depends
- Unimportant
- Should not be included

* 35. The first aider should know that people with an intellectual disability may use a range of communication methods, e.g. they may rely more on non-verbal communication or communication aids.

- Essential
- Important
- Don't know/depends
- Unimportant
- Should not be included

* 36. The first aider should try to use any communication aids that the person uses.

- Essential
- Important
- Don't know/depends
- Unimportant
- Should not be included

* 37. If the first aider is using writing to communicate with the person, they should use larger text or bullet points.

- Essential
- Important
- Don't know/depends
- Unimportant
- Should not be included

* 38. The first aider should know that the person may need assistance to express themselves verbally.

- Essential
- Important
- Don't know/depends
- Unimportant
- Should not be included

* 39. The first aider should speak to the person the same way they would speak to others of that age.

- Essential
- Important
- Don't know/depends
- Unimportant
- Should not be included

* 40. The first aider should use simple, short statements or questions.

- Essential
- Important
- Don't know/depends
- Unimportant
- Should not be included

* 41. The first aider should use descriptive rather than technical language.

- Essential
- Important
- Don't know/depends
- Unimportant
- Should not be included

* 42. The first aider should not use abbreviations when talking to the person, e.g. "CBT".

- Essential
- Important
- Don't know/depends
- Unimportant
- Should not be included

* 43. The first aider should not use metaphors, sarcasm or idioms (e.g. "under the weather"), as the person may misunderstand these.

- Essential
- Important
- Don't know/depends
- Unimportant
- Should not be included

* 44. The first aider should not change their style of communication unless the person asks them to, e.g. speaking louder or slower.

- Essential
- Important
- Don't know/depends
- Unimportant
- Should not be included

* 45. The first aider should not speak louder or slower than usual, in an attempt to aid the person's comprehension.

- Essential
- Important
- Don't know/depends
- Unimportant
- Should not be included

* 46. The first aider should try to match their vocabulary usage to the person's, e.g. if the person only speaks with simple words, the first aider should also use simple words.

- Essential
- Important
- Don't know/depends
- Unimportant
- Should not be included

* 47. The first aider should try to establish appropriate physical boundaries that both they and the person are comfortable with.

- Essential
- Important
- Don't know/depends
- Unimportant
- Should not be included

* 48. The first aider should be aware that some people with an intellectual disability may respond with an unexpected physical response, e.g. hugging or kissing new people.

- Essential
- Important
- Don't know/depends
- Unimportant
- Should not be included

* 49. If the person is using stimming behaviours or other distractions to manage their emotions during the conversation, the first aider should not attempt to stop them.

- Essential
- Important
- Don't know/depends
- Unimportant
- Should not be included

* 50. If the person is distressed, the first aider should try to make the environment less stressful for them (e.g. turning off the TV or asking people to be quiet) as the person may be responding to overstimulation.

- Essential
- Important
- Don't know/depends
- Unimportant
- Should not be included

51. Please provide any additional items or comments related to this section.

Development of guidelines for considerations when providing mental health first aid to a person with an intellectual disability

COMMUNICATING WITH THE PERSON cont.

Please rate how important (from ‘essential’ to ‘should not be included’) you think it is that each statement be included in the guidelines.

Please keep our definitions in mind when responding to this section. You can access the definitions here.

Comprehension

* 52. The first aider should reinforce important messages to increase the likelihood the person will understand and remember.

- Essential
- Important
- Don't know/depends
- Unimportant
- Should not be included

* 53. The first aider should give the person time to think about what has been said or to express themselves.

- Essential
- Important
- Don't know/depends
- Unimportant
- Should not be included

* 54. The first aider should not assume the person cannot understand them.

- Essential
- Important
- Don't know/depends
- Unimportant
- Should not be included

* 55. The first aider should not assume that the person's ability to express themseves is an indication of how much they understand.

- Essential
- Important
- Don't know/depends
- Unimportant
- Should not be included

* 56. The first aider should not pretend to understand the person if they do not.

- Essential
- Important
- Don't know/depends
- Unimportant
- Should not be included

* 57. The first aider should not assume that the person will tell them if they do not understand.

- Essential
- Important
- Don't know/depends
- Unimportant
- Should not be included

* 58. As people with an intellectual disability may pretend they understand when they do not, the first aider should tell the person that it is ok to say if they don't understand, e.g. by saying "I need to make sure I explain it properly. Please let me know if I am not clear enough."

- Essential
- Important
- Don't know/depends
- Unimportant
- Should not be included

* 59. If, when the first aider asks about when something occurred, or how long something has been happening, the person struggles to explain in terms of dates, times, hours or weeks, the first aider should ask in a way that makes it meaningful for them, e.g. “Have you felt this way since before your birthday, or after?” or “Was that at dinner time or breakfast time?”

- Essential
- Important
- Don't know/depends
- Unimportant
- Should not be included

* 60. If the first aider thinks the person has lost track of the conversation, they should repeat what they have said.

- Essential
- Important
- Don't know/depends
- Unimportant
- Should not be included

* 61. If the first aider thinks the person has lost track of the conversation, they should try to express it in a different way.

- Essential
- Important
- Don't know/depends
- Unimportant
- Should not be included

* 62. The first aider should allow the person talk about their experiences.

- Essential
- Important
- Don't know/depends
- Unimportant
- Should not be included

* 63. The first aider should try to help the person identify and label what they are feeling, e.g. frustrated.

- Essential
- Important
- Don't know/depends
- Unimportant
- Should not be included

* 64. The first aider should not assume that the person can't describe their symptoms of mental health problems.

- Essential
- Important
- Don't know/depends
- Unimportant
- Should not be included

* 65. The first aider should be aware that they may not be correctly interpreting the person's expressed emotions, e.g. if the person says that they are always bored it may be that they are having trouble experiencing pleasure.

- Essential
- Important
- Don't know/depends
- Unimportant
- Should not be included

* 66. The first aider should be aware that the person may describe their emotional symptoms using physical descriptions, e.g. their heart hurts or they are sad in their stomach.

- Essential
- Important
- Don't know/depends
- Unimportant
- Should not be included

67. Please provide any additional items or comments related to this section.

Development of guidelines for considerations when providing mental health first aid to a person with an intellectual disability

COMMUNICATING WITH THE PERSON cont.

Please rate how important (from ‘essential’ to ‘should not be included’) you think it is that each statement be included in the guidelines.

Please keep our definitions in mind when responding to this section. You can access the definitions here.

Questions

* 68. The first aider should not try to finish the person's sentences.

- Essential
- Important
- Don't know/depends
- Unimportant
- Should not be included

* 69. The first aider should not ask the person probing questions about their disability.

- Essential
- Important
- Don't know/depends
- Unimportant
- Should not be included

* 70. The first aider should not ask other people probing questions about the person's disability.

- Essential
- Important
- Don't know/depends
- Unimportant
- Should not be included

* 71. The first aider should start by asking the person questions they know the person can answer, as this can help to reduce the person's anxiety and build confidence.

- Essential
- Important
- Don't know/depends
- Unimportant
- Should not be included

* 72. If the first aider asks the person a question, they should wait for a response before asking a second question.

- Essential
- Important
- Don't know/depends
- Unimportant
- Should not be included

* 73. The first aider should avoid asking the person leading questions (e.g. "You're feeling sad aren't you?") as this may influence them to respond with what they think the first aider wants to hear or what they think is the 'right' answer.

- Essential
- Important
- Don't know/depends
- Unimportant
- Should not be included

* 74. If the first aider asks the person a question that offers a choice of answers, they should be aware that the person might choose the last option and should therefore check this by asking the question again later in a different way.

- Essential
- Important
- Don't know/depends
- Unimportant
- Should not be included

* 75. The first aider should not provide the person with too many options as this may be confusing for them.

- Essential
- Important
- Don't know/depends
- Unimportant
- Should not be included

76. Please provide any additional items or comments related to this section.

Development of guidelines for considerations when providing mental health first aid to a person with an intellectual disability

COMMUNICATING WITH THE PERSON cont.

Please rate how important (from 'essential' to 'should not be included') you think it is that each statement be included in the guidelines.

Please keep our definitions in mind when responding to this section. You can access the definitions here.

Engaging others in the conversation

* 77. If the first aider is having difficulty understanding the person, they should ask the person if there is anyone they would like to be part of the conversation who could help the first aider understand.

- ◯ Essential
- ◯ Important
- ◯ Don't know/depends
- ◯ Unimportant
- ◯ Should not be included

* 78. The first aider should ask the person if there is anyone they would like to be part of the conversation to support them and make them feel at ease, e.g. a family member or friend.

- ◯ Essential
- ◯ Important
- ◯ Don't know/depends
- ◯ Unimportant
- ◯ Should not be included

* 79. The first aider should be aware that if other people are present, the person may feel pressured and less able to speak openly or make their own decisions.

- ◯ Essential
- ◯ Important
- ◯ Don't know/depends
- ◯ Unimportant
- ◯ Should not be included

* 80. The first aider should direct all communication to the person, even if there are other people present.

- ◯ Essential
- ◯ Important
- ◯ Don't know/depends
- ◯ Unimportant
- ◯ Should not be included

* 81. If the first aider has clarified what the person has communicated with a carer or support person, the first aider should check with the person if the correct information has been relayed.

- Essential
- Important
- Don't know/depends
- Unimportant
- Should not be included

82. Please provide any additional items or comments related to this section.

Development of guidelines for considerations when providing mental health first aid to a person with an intellectual disability

RESPECT AND RIGHTS

This section contains statements about what the first aider needs to know about respecting the person and their rights.

Please rate how important (from ‘essential’ to ‘should not be included’) you think it is that each statement be included in the guidelines.

Please keep our definitions in mind when responding to this section. You can access the definitions here.

Respecting the person and their rights

* 83. The first aider should use 'person-first language' when referring to or talking about the person, e.g. "a person with an intellectual disability" rather than "an intellectually disabled person".

- Essential
- Important
- Don't know/depends
- Unimportant
- Should not be included

* 84. The first aider should know that a person with an intellectual disability has as much right to make decisions for themselves as anyone else.

- Essential
- Important
- Don't know/depends
- Unimportant
- Should not be included

* 85. The first aider should not assume that they know what is best for the person.

- Essential
- Important
- Don't know/depends
- Unimportant
- Should not be included

* 86. The first aider should know that the person's right to privacy should not be waived simply because they have an intellectual disability.

- Essential
- Important
- Don't know/depends
- Unimportant
- Should not be included

* 87. If the first aider needs to ask someone else for information, they should get the person's permission before doing so.

- Essential
- Important
- Don't know/depends
- Unimportant
- Should not be included

* 88. If the first aider needs to share information about the person with anyone else, they should get the person's permission before doing so.

- Essential
- Important
- Don't know/depends
- Unimportant
- Should not be included

* 89. The first aider should not share information with others without the person's permission, unless there is a risk of harm to the person or others.

- Essential
- Important
- Don't know/depends
- Unimportant
- Should not be included

* 90. The first aider should respect the person's autonomy while considering the extent to which they are able to make decisions for themselves.

- Essential
- Important
- Don't know/depends
- Unimportant
- Should not be included

* 91. The first aider should not involve another person in the first aid without the person's permission, unless there is risk of harm to the person or others.

- Essential
- Important
- Don't know/depends
- Unimportant
- Should not be included

* 92. The first aider should try to find out if the person has a legal guardian or conservator appointed.

- Essential
- Important
- Don't know/depends
- Unimportant
- Should not be included

* 93. If the person has a legal guardian or conservator appointed, the first aider should contact them before offering mental health first aid, unless the person is at risk of harm to themselves or others.

- Essential
- Important
- Don't know/depends
- Unimportant
- Should not be included

94. Please provide any additional items or comments related to this section.

Development of guidelines for considerations when providing mental health first aid to a person with an intellectual disability

BEING SUPPORTIVE

This section contains statements about what the first aider needs to know about being supportive to a person who may be experiencing mental health problems.

Please rate how important (from ‘essential’ to ‘should not be included’) you think it is that each statement be included in the guidelines.

Please keep our definitions in mind when responding to this section. You can access the definitions here.

Being supportive

* 95. The first aider should ask the person how they would like to be supported.

- Essential
- Important
- Don't know/depends
- Unimportant
- Should not be included

* 96. The first aider should not make assumptions about what kind of support the person may need.

- Essential
- Important
- Don't know/depends
- Unimportant
- Should not be included

* 97. The first aider should ask the person if they would like some information about mental health problems and in what form they would like the information.

- Essential
- Important
- Don't know/depends
- Unimportant
- Should not be included

* 98. The first aider should ask the person if they would like any practical assistance with tasks while being careful not to take over or encourage dependency.

- Essential
- Important
- Don't know/depends
- Unimportant
- Should not be included

* 99. The first aider should not adopt an over-involved or over-protective attitude toward the person.

- Essential
- Important
- Don't know/depends
- Unimportant
- Should not be included

* 100. The first aider should ask the person if they need support to use coping strategies for their mental health problems.

- Essential
- Important
- Don't know/depends
- Unimportant
- Should not be included

101. Please provide any additional items or comments related to this section.

Development of guidelines for considerations when providing mental health first aid to a person with an intellectual disability

PSYCHOSIS

This section contains statements about what the first aider needs to know if they are concerned the person may be experiencing psychosis.

Please rate how important (from ‘essential’ to ‘should not be included’) you think it is that each statement be included in the guidelines.

Please keep our definitions in mind when responding to this section. You can access the definitions here.

Psychosis

* 102. If the person thinks they are being controlled by others, the first aider should not assume this is a delusion, as people with an intellectual disability may have less control over their lives.

- Essential
- Important
- Don't know/depends
- Unimportant
- Should not be included

* 103. If the person appears to be experiencing hallucinations or delusions, the first aider should not assume that this is the result of a mental health problem, as it may be the actual reality of the person, e.g. they may report that others are staring at them which could be due to their 'different' appearance or they may believe a carer is trying to hurt them if they don't get on with that person.

- Essential
- Important
- Don't know/depends
- Unimportant
- Should not be included

* 104. If the person appears to be experiencing hallucinations or delusions that are not causing them distress or putting them in danger, the first aider should not attempt to intervene.

- Essential
- Important
- Don't know/depends
- Unimportant
- Should not be included

* 105. If the person is talking to themselves, the first aider should not assume they are experiencing hallucinations, as it is not uncommon for people with an intellectual disability to talk to themselves, have a conversation with an imaginary person or inanimate object, repeat conversations or replay arguments they have heard.

- Essential
- Important
- Don't know/depends
- Unimportant
- Should not be included

106. Please provide any additional items or comments related to this section.

ANXIETY

This section contains statements about what the first aider needs to know if they are concerned the person may be experiencing anxiety.

Please rate how important (from ‘essential’ to ‘should not be included’) you think it is that each statement be included in the guidelines.

Please keep our definitions in mind when responding to this section. You can access the definitions here.

Anxiety

* 107. The first aider should know that anxiety and stress disorders in people with an intellectual disability are often misdiagnosed as 'challenging behaviour'.

- Essential
- Important
- Don't know/depends
- Unimportant
- Should not be included

* 108. The first aider should be aware that in people with an intellectual disability obsessive compulsive disorder is often confused with special interests or unique routines, e.g. someone may be obsessed with a TV character and love to read or hear about them or want to eat off a certain plate.

- Essential
- Important
- Don't know/depends
- Unimportant
- Should not be included

109. Please provide any additional items or comments related to this section.

SELF-INJURY

This section contains statements about what the first aider needs to know if they are concerned the person may be injuring themselves.

Please rate how important (from 'essential' to 'should not be included') you think it is that each statement be included in the guidelines.

Please keep our definitions in mind when responding to this section. You can access the definitions here.

Self-injury

* 110. The first aider should be aware that people with an intellectual disability may injure themselves for a range of reasons, e.g. to communicate a need such as hunger, to manage distress (NSSI), or as a result of stimming behaviours.

- Essential
- Important
- Don't know/depends
- Unimportant
- Should not be included

* 111. The first aider should not dismiss self-injurious behaviours as stimming or 'challenging behaviour', as these may be non-suicidal self-injury motivated by distress.

- Essential
- Important
- Don't know/depends
- Unimportant
- Should not be included

* 112. As the person may be self-injuring because they are trying to communicate something or have a particular need (e.g. hunger, physical pain, or they don't like what is on the TV), the first aider should try to find out what they are trying to communicate or what they need.

- Essential
- Important
- Don't know/depends
- Unimportant
- Should not be included

* 113. If the person is self-injurying, the first aider should:

	Essential	Important	Don't know/depends	Unimportant	Should not be included
try to physically restrain the person.	◯	◯	◯	◯	◯
not try to physically restrain the person.	◯	◯	◯	◯	◯
seek assistance	◯	◯	◯	◯	◯
try to minimise the person's risk of immediate harm, e.g. putting a pillow between the person's head and a wall.	◯	◯	◯	◯	◯
know that these can help the person to feel calm and should not be viewed as self-injurious behaviour.	◯	◯	◯	◯	◯

* 114. If the person is engaging in stimming behaviour that is not harmful, the first aider should know that these can help the person to feel calm and should not be viewed as self-injurious behaviour.

◯ Essential

◯ Important

◯ Don't know/depends

◯ Unimportant

◯ Should not be included

115. Please provide any additional items or comments related to this section.

Development of guidelines for considerations when providing mental health first aid to a person with an intellectual disability

SUBSTANCE USE

This section contains statements about what the first aider needs to know if they are concerned the person may be misusing substances.

Please rate how important (from ‘essential’ to ‘should not be included’) you think it is that each statement be included in the guidelines.

Please keep our definitions in mind when responding to this section. You can access the definitions here.

Substance use

* 116. If the first aider is concerned the person may be using alcohol or other drugs, they should not delay approaching them about their concerns, because people with an intellectual disability who use alcohol or other drugs are more likely to misuse them and experience negative consequences as a result.

- Essential
- Important
- Don't know/depends
- Unimportant
- Should not be included

* 117. The first aider should ask the person about their substance use (e.g. what, how much, for how long), rather than using clinical terms such as 'substance misuse' or 'substance abuse'.

- Essential
- Important
- Don't know/depends
- Unimportant
- Should not be included

* 118. If the person appears to be incoherent, physically unsteady, confused, disoriented or frightened, the first aider should not assume that the person has been using alcohol or other drugs.

- Essential
- Important
- Don't know/depends
- Unimportant
- Should not be included

* 119. If the first aider thinks the person may have been using alcohol or other drugs, they should try to find out by asking them in a way that does not imply that they have been, e.g. by asking what the person has been doing today.

- Essential
- Important
- Don't know/depends
- Unimportant
- Should not be included

* 120. The first aider should be aware that the person may have difficulty understanding the consequences of their substance use.

- Essential
- Important
- Don't know/depends
- Unimportant
- Should not be included

* 121. If the person does not seem to understand the consequences of their substance use, the first aider should provide information about these.

- Essential
- Important
- Don't know/depends
- Unimportant
- Should not be included

* 122. If the person is taking prescribed medication, first aider should explain to them that mixing medications with alcohol or other drugs can be dangerous.

- Essential
- Important
- Don't know/depends
- Unimportant
- Should not be included

* 123. The first aider should know that the person may be susceptible to peer pressure to use substances.

- Essential
- Important
- Don't know/depends
- Unimportant
- Should not be included

* 124. The first aider should not dismiss concerns about the person's substance use based on the quantity they are using, as people with an intellectual disability may experience stronger effects from smaller quantities.

- Essential
- Important
- Don't know/depends
- Unimportant
- Should not be included

* 125. The first aider should not dismiss the person's substance use as understandable in their circumstances, e.g. people may think "I would drink too if I were them."

- Essential
- Important
- Don't know/depends
- Unimportant
- Should not be included

* 126. If the person thinks a support group may be helpful, the first aider should offer to support them to contact the organisers of the group to explore if it is appropriate for them.

- Essential
- Important
- Don't know/depends
- Unimportant
- Should not be included

* 127. The first aider should suggest to the person that they use an online screening tool for substance use problems.

- Essential
- Important
- Don't know/depends
- Unimportant
- Should not be included

* 128. If the person wants to use an online screening tool for substance use problems, the first aider should offer to assist them or find someone who can.

- Essential
- Important
- Don't know/depends
- Unimportant
- Should not be included

129. Please provide any additional items or comments related to this section.

EATING DISORDERS

This section contains statements about what the first aider needs to know if they are concerned the person may have an eating disorder.

Please rate how important (from ‘essential’ to ‘should not be included’) you think it is that each statement be included in the guidelines.

Please keep our definitions in mind when responding to this section. You can access the definitions here.

Eating disorders

* 130. The first aider should not dismiss signs of a potential eating disorder as eating rituals related to the person's intellectual disability.

- Essential
- Important
- Don't know/depends
- Unimportant
- Should not be included

131. Please provide any additional items or comments related to this section.

Development of guidelines for considerations when providing mental

DEMENTIA

This section contains statements about what the first aider needs to know if they are concerned the person may be developing dementia.

Please rate how important (from ‘essential’ to ‘should not be included’) you think it is that each statement be included in the guidelines.

Please keep our definitions in mind when responding to this section. You can access the definitions here.

Dementia

* 132. If the first aider is concerned that the person may be experiencing confusion related to dementia, they should ask the person if they can raise their concerns with the person's carer or support person.

- Essential
- Important
- Don't know/depends
- Unimportant
- Should not be included

* 133. If the first aider is concerned that the person may be experiencing confusion related to dementia and they are concerned for the person's safety, they should raise their concerns with the person's carer or support person.

- Essential
- Important
- Don't know/depends
- Unimportant
- Should not be included

134. Please provide any additional items or comments related to this section.

Development of guidelines for considerations when providing mental health first aid to a person with an intellectual disability

ABUSE

This section contains statements about what the first aider needs to know if they are concerned the person may be experiencing abuse.

Please rate how important (from ‘essential’ to ‘should not be included’) you think it is that each statement be included in the guidelines.

Please keep our definitions in mind when responding to this section. You can access the definitions here.

Abuse

* 135. The first aider should know that people with intellectual disabilities are at greater risk of abuse and neglect, and are more likely to experience abuse that is more severe, repeated, and for a longer period of time, than the general population.

- Essential
- Important
- Don't know/depends
- Unimportant
- Should not be included

* 136. The first aider should know that the person might not be aware that any abuse they may be experiencing is unacceptable.

- Essential
- Important
- Don't know/depends
- Unimportant
- Should not be included

* 137. The first aider should know that people with an intellectual disability are more likely than the general population to be abused by a caregiver or someone they know.

- Essential
- Important
- Don't know/depends
- Unimportant
- Should not be included

* 138. The first aider should try to talk to the person without the presence of others who may influence their ability to speak freely.

- Essential
- Important
- Don't know/depends
- Unimportant
- Should not be included

* 139. If the person discloses to the first aider that they have been abused, the first aider should give the person information about advocacy services.

- Essential
- Important
- Don't know/depends
- Unimportant
- Should not be included

* 140. The first aider should know the local laws regarding mandatory reporting of abuse.

- Essential
- Important
- Don't know/depends
- Unimportant
- Should not be included

* 141. If the first aider has reason to believe the person may be experiencing abuse, they should encourage the person to report this to an appropriate service, e.g. the police, disability abuse and neglect hotline, crisis support service.

- Essential
- Important
- Don't know/depends
- Unimportant
- Should not be included

* 142. If the first aider has reason to believe the person may be experiencing abuse and is in immediate danger, they should encourage the person to call the police.

- Essential
- Important
- Don't know/depends
- Unimportant
- Should not be included

* 143. If the first aider has reason to believe the person may be experiencing abuse and is in immediate danger, and the person is unable to call the police, the first aider should call on their behalf.

- Essential
- Important
- Don't know/depends
- Unimportant
- Should not be included

* 144. If the first aider has reason to believe the person may be experiencing abuse and is in immediate danger, they should call the police.

- Essential
- Important
- Don't know/depends
- Unimportant
- Should not be included

* 145. If the first aider has reason to believe the person may be experiencing abuse, they should contact an appropriate service for advice, e.g. disability abuse and neglect hotline, crisis support service.

- Essential
- Important
- Don't know/depends
- Unimportant
- Should not be included

146. Please provide any additional items or comments related to this section.

PLEASE NOTE:
The following statement applies **only** to situations in which the first aider is a **paid disability worker.**
Please rate how important (from ‘essential’ to ‘should not be included’) you think it is that the statement be included in the guidelines when the **first aider is a paid disability worker.**

* 147. If the person discloses that they have been abused, the first aider should record the exact facts about what they have been told and have observed, with the person's permission.

- Essential
- Important
- Don't know/depends
- Unimportant
- Should not be included

148. Please provide any additional items or comments related to this section.

Development of guidelines for considerations when providing mental health first aid to a person with an intellectual disability

TRAUMA

This section contains statements about what the first aider needs to know if they are concerned the person may be experiencing trauma.

Please rate how important (from ‘essential’ to ‘should not be included’) you think it is that each statement be included in the guidelines.

Please keep our definitions in mind when responding to this section. You can access the definitions here.

Trauma

* 149. The first aider should know that people with an intellectual disability are more likely to have experienced trauma than the general population.

- Essential
- Important
- Don't know/depends
- Unimportant
- Should not be included

* 150. The first aider should know that seemingly 'less significant' events can be traumatic for a person with an intellectual disability, e.g. repeated rejection, living in situations where they lack control, teasing and name calling, having extended hospitalisations.

- Essential
- Important
- Don't know/depends
- Unimportant
- Should not be included

* 151. The first aider should be aware that just because the person is not avoiding certain situations, this does not mean it is not traumatic for them as they may not be able to remove themselves from this situation.

- Essential
- Important
- Don't know/depends
- Unimportant
- Should not be included

152. Please provide any additional items or comments related to this section.

OTHER SUPPORTS

This section contains statements about what the first aider needs to know about encouraging other supports.

Please rate how important (from ‘essential’ to ‘should not be included’) you think it is that each statement be included in the guidelines.

Please keep our definitions in mind when responding to this section. You can access the definitions here.

Other supports

* 153. If the person needs additional support, but family and friends are under stress or 'burnt out', the first aider should assist the person to find alternate supports.

- Essential
- Important
- Don't know/depends
- Unimportant
- Should not be included

* 154. If the first aider is going to suggest self-help strategies, they should consider the person's ability to engage in these strategies.

- Essential
- Important
- Don't know/depends
- Unimportant
- Should not be included

* 155. If the person wants to use self-help strategies but need support to do so, the first aider should suggest a disability-specific service (where available) that offers specialised education programs to assist them to learn these skills.

- Essential
- Important
- Don't know/depends
- Unimportant
- Should not be included

* 156. The first aider should ask the person if they have used self-help strategies in the past that they found helpful, and if so, support them to use these if needed.

- Essential
- Important
- Don't know/depends
- Unimportant
- Should not be included

157. Please provide any additional items or comments related to this section.

DIFFICULTIES THE FIRST AIDER MAY ENCOUNTER

This section contains statements about what a first aider should know and do regarding difficulties they may encounter.

Please rate how important (from ‘essential’ to ‘should not be included’) you think it is that each statement be included in the guidelines.

Please keep our definitions in mind when responding to this section. You can access the definitions here.

There are three parts to this section:
1. General
2. Aggressive behaviours
3. Sexually inappropriate behaviours

General

* 158. If the person is exhibiting behaviours that the first aider finds challenging, the first aider should ask the person what they are feeling.

- Essential
- Important
- Don't know/depends
- Unimportant
- Should not be included

* 159. The first aider should know that symptoms of a mental health problem can contribute to an increase in behaviours that others find challenging.

- Essential
- Important
- Don't know/depends
- Unimportant
- Should not be included

* 160. The first aider should know that behaviours that others find challenging may indirectly contribute to mental health problems, e.g. these behaviours could lead to increased isolation which can contribute to low mood.

- Essential
- Important
- Don't know/depends
- Unimportant
- Should not be included

* 161. The first aider should know that a decrease in behaviours that others find challenging could indicate that the person is experiencing mental health problems.

- Essential
- Important
- Don't know/depends
- Unimportant
- Should not be included

* 162. If the person is exhibiting behaviours that others find challenging, the first aider should not assume that the person is experiencing mental health problems.

- Essential
- Important
- Don't know/depends
- Unimportant
- Should not be included

* 163. If the person is exhibiting behaviours that the first aider finds challenging, the first aider should explore the reasons for these, e.g. ask if they are in pain or hungry, ask if there is something that is bothering them, look for overwhelming environmental factors (such as bright lights).

- Essential
- Important
- Don't know/depends
- Unimportant
- Should not be included

* 164. If the person is exhibiting behaviours that the first aider finds challenging, they should ask them to limit their behaviour, while validating their feelings, e.g. "I can see you're really upset and that's okay, but please stop hitting yourself".

- Essential
- Important
- Don't know/depends
- Unimportant
- Should not be included

* 165. If the person is exhibiting behaviours that the first aider finds challenging, the first aider should know that trying to stop or control the behaviour without addressing how the person is feeling is not likely to be successful.

- Essential
- Important
- Don't know/depends
- Unimportant
- Should not be included

* 166. The first aider should encourage the person to express their feelings through verbal communication or communication tools instead of through the behaviour that the first aider is finding challenging.

- Essential
- Important
- Don't know/depends
- Unimportant
- Should not be included

* 167. the person is exhibiting behaviours that others find challenging, the first aider should encourage them to seek appropriate professional help.

- Essential
- Important
- Don't know/depends
- Unimportant
- Should not be included

* 168. The first aider should know that behaviours that others find challenging can have different functions for different people, e.g. pacing may indicate boredom in one person and a mental health problem in another.

- Essential
- Important
- Don't know/depends
- Unimportant
- Should not be included

169. Please provide any additional items or comments related to this section.

DIFFICULTIES THE FIRST AIDER MAY ENCOUNTER cont.

Please rate how important (from ‘essential’ to ‘should not be included’) you think it is that each statement be included in the guidelines.

Please keep our definitions in mind when responding to this section. You can access the definitions here.

Aggressive behaviours

* 170. If the person is being aggressive, the first aider should only use physical force as a last resort to prevent injury to the person or others.

- ○ Essential
- ○ Important
- ○ Don't know/depends
- ○ Unimportant
- ○ Should not be included

171. Please provide any additional items or comments related to this section.

Development of guidelines for considerations when providing mental health first aid to a person with an intellectual disability

DIFFICULTIES THE FIRST AIDER MAY ENCOUNTER cont.

Please rate how important (from ‘essential’ to ‘should not be included’) you think it is that each statement be included in the guidelines.

Please keep our definitions in mind when responding to this section. You can access the definitions here.

Sexually inappropriate behaviours

* 172. The first aider should be aware that sexually inappropriate behaviour may indicate that the person is trying to communicate that they are experiencing abuse.

- Essential
- Important
- Don't know/depends
- Unimportant
- Should not be included

* 173. The first aider should know that if the person is exhibiting inappropriate sexual behaviours, this could indicate that the person is experiencing a mental health problem.

- Essential
- Important
- Don't know/depends
- Unimportant
- Should not be included

* 174. If the person is exhibiting sexually inappropriate behaviour the first aider should tell the person that the behaviours are not acceptable.

- Essential
- Important
- Don't know/depends
- Unimportant
- Should not be included

* 175. If the person is exhibiting sexually inappropriate behaviour that could be considered sexual or physical assault, the first aider should call the police.

- Essential
- Important
- Don't know/depends
- Unimportant
- Should not be included

176. Please provide any additional items or comments related to this section.

PROFESSIONAL HELP

This section contains statements about what the first aider needs to know and do when encouraging the person to seek professional help.

Please rate how important (from 'essential' to 'should not be included') you think it is that each statement be included in the guidelines.

Please keep our definitions in mind when responding to this section. You can access the definitions here.

There are three parts to this section:

1. Professional help
2. Supporting the person to attend a professional appointment
3. If the person has an appointment with a professional

Professional help

* 177. As some symptoms of mental health problems may be explained by physical conditions, the first aider should encourage the person to seek medical advice.

- Essential
- Important
- Don't know/depends
- Unimportant
- Should not be included

* 178. As some symptoms of mental health problems may be explained by medication side effects, the first aider should encourage the person to seek medical advice.

- Essential
- Important
- Don't know/depends
- Unimportant
- Should not be included

* 179. The first aider should explain to the person their options for seeking professional help.

- Essential
- Important
- Don't know/depends
- Unimportant
- Should not be included

* 180. The first aider should provide the person with information that may help them to make a decision about seeking professional help, e.g. what is involved in the various options and the benefits of each option.

- Essential
- Important
- Don't know/depends
- Unimportant
- Should not be included

* 181. The first aider should know that people with intellectual disability are entitled to use the same mental health services as the general population.

- Essential
- Important
- Don't know/depends
- Unimportant
- Should not be included

* 182. The first aider should find out if there are locally available mental health services that have expertise in intellectual disability.

- Essential
- Important
- Don't know/depends
- Unimportant
- Should not be included

* 183. The first aider should tell the person about locally available mental health services that have expertise in intellectual disability, where these are available.

- Essential
- Important
- Don't know/depends
- Unimportant
- Should not be included

* 184. The first aider should know that the person may not want to attend a mental health service that specialises in intellectual disability.

- Essential
- Important
- Don't know/depends
- Unimportant
- Should not be included

* 185. The first aider should provide the person with information about seeking professional help in a way that supports the person to make an informed choice, e.g that the information is understood and meaningful.

- Essential
- Important
- Don't know/depends
- Unimportant
- Should not be included

* 186. The first aider should know that there may not be appropriate resources about professional help-seeking available for people with intellectual disability.

- Essential
- Important
- Don't know/depends
- Unimportant
- Should not be included

* 187. The first aider should be aware that the person may not be used to having a choice and should let the person know that they can make their own decisions about seeking professional help and can change their mind at any time.

- Essential
- Important
- Don't know/depends
- Unimportant
- Should not be included

* 188. The first aider should know that the person has the right to accept or refuse professional help.

- Essential
- Important
- Don't know/depends
- Unimportant
- Should not be included

* 189. The first aider should respect the person's right to make their own decisions regarding seeking professional help.

- Essential
- Important
- Don't know/depends
- Unimportant
- Should not be included

190. Please provide any additional items or comments related to this section.

Development of guidelines for considerations when providing mental health first aid to a person with an intellectual disability

PROFESSIONAL HELP cont.

This section contains statements about what the first aider needs to know and do when supporting the person to attend a professional appointment.

Please rate how important (from ‘essential’ to ‘should not be included’) you think it is that each statement be included in the guidelines.

Please keep our definitions in mind when responding to this section. You can access the definitions here.

Supporting the person to attend an appointment

* 191. The first aider should offer the person the support they require to make an appointment with a professional.

- Essential
- Important
- Don't know/depends
- Unimportant
- Should not be included

* 192. If the first aider thinks the person needs support to access professional help, they should ask the person if there is someone they know well and trust who they would like to support them.

- Essential
- Important
- Don't know/depends
- Unimportant
- Should not be included

* 193. The first aider should be aware of the barriers that may prevent the person from seeking professional help.

- Essential
- Important
- Don't know/depends
- Unimportant
- Should not be included

* 194. The first aider should try to find out if there are specific barriers for the person in accessing professional help and try to help them overcome these, e.g. accessing transportation.

- Essential
- Important
- Don't know/depends
- Unimportant
- Should not be included

* 195. If appropriate to the relationship, the first aider should support the person to make an appointment with a health professional.

- ◯ Essential
- ◯ Important
- ◯ Don't know/depends
- ◯ Unimportant
- ◯ Should not be included

* 196. If appropriate to the relationship, the first aider should offer to make the person an appointment with a health professional.

- ◯ Essential
- ◯ Important
- ◯ Don't know/depends
- ◯ Unimportant
- ◯ Should not be included

* 197. If appropriate to the relationship, the first aider should support the person to attend an appointment with a health professional.

- ◯ Essential
- ◯ Important
- ◯ Don't know/depends
- ◯ Unimportant
- ◯ Should not be included

* 198. If appropriate to the relationship, the first aider should offer to accompany the person to an appointment with a health professional.

- ◯ Essential
- ◯ Important
- ◯ Don't know/depends
- ◯ Unimportant
- ◯ Should not be included

* 199. If challenges arise that prevent the person from receiving appropriate professional help, the first aider should try to find an advocate who can support the person to access appropriate help.

- Essential
- Important
- Don't know/depends
- Unimportant
- Should not be included

* 200. The first aider should advise the person to book a longer professional appointment to allow enough time for a thorough assessment.

- Essential
- Important
- Don't know/depends
- Unimportant
- Should not be included

* 201. If the person wants additional support when seeking professional help, the first aider should encourage them to use a personal advocate.

- Essential
- Important
- Don't know/depends
- Unimportant
- Should not be included

202. Please provide any additional items or comments related to this section.

PROFESSIONAL HELP cont.

This section contains statements about what the first aider needs to know and do if the person has an appointment with a professional.

Please rate how important (from ‘essential’ to ‘should not be included’) you think it is that each statement be included in the guidelines.

Please keep our definitions in mind when responding to this section. You can access the definitions here.

If the person has an appointment with a professional

* 203. The first aider should explain to the person what they may expect in their appointment.

- Essential
- Important
- Don't know/depends
- Unimportant
- Should not be included

* 204. The first aider should help the person to prepare for their appointment with a health professional, e.g. gather their health information, write a list of questions to ask.

- Essential
- Important
- Don't know/depends
- Unimportant
- Should not be included

* 205. The first aider should help the person to anticipate the upcoming appointment by making sure they know the time and place, and who they will see.

- Essential
- Important
- Don't know/depends
- Unimportant
- Should not be included

* 206. If possible, the first aider should try to arrange for a professional assessment to be conducted in an environment the person is familiar with (e.g. their own home).

- Essential
- Important
- Don't know/depends
- Unimportant
- Should not be included

* 207. If the person thinks they are in trouble because they are going to see a professional, the first aider should reassure the person that they are not.

- Essential
- Important
- Don't know/depends
- Unimportant
- Should not be included

* 208. If the first aider supports the person to attend a professional appointment, they should let the health professional know if the person has any specific fears or needs that may affect the appointment, with the person's permission.

- Essential
- Important
- Don't know/depends
- Unimportant
- Should not be included

* 209. If the first aider supports the person to attend a professional appointment, they should explain to the health professional the person's communication style and preferences, with the person's permission.

- Essential
- Important
- Don't know/depends
- Unimportant
- Should not be included

* 210. If the first aider accompanies the person to an appointment, they should assist the professional to communicate and interact with the person, with the person's permission.

- Essential
- Important
- Don't know/depends
- Unimportant
- Should not be included

* 211. If the first aider accompanies the person to an appointment and the professional is having difficultly understanding the person, the first aider should assist, with the person's permission.

- Essential
- Important
- Don't know/depends
- Unimportant
- Should not be included

212. Please provide any additional items or comments related to this section.

Development of guidelines for considerations when providing mental health first aid to a person with an intellectual disability

CRISIS SITUATION

This section contains statements about what the first aider needs to know and do if the person is in crisis.

Please rate how important (from ‘essential’ to ‘should not be included’) you think it is that each statement be included in the guidelines.

Please keep our definitions in mind when responding to this section. You can access the definitions here.

There are two parts to this section:

1. Crisis - general
2. Suicide

Crisis - general

* 213. The first aider should ask the person if there are certain objects or activities that they find comforting and might help the person maintain calm.

- Essential
- Important
- Don't know/depends
- Unimportant
- Should not be included

* 214. The first aider should check if the person is wearing a medical alert bracelet or pendant, or has a medical alert tattoo.

- Essential
- Important
- Don't know/depends
- Unimportant
- Should not be included

* 215. If the first aider calls a mental health crisis team, they should describe the person's symptoms and behaviour and let them know the person has an intellectual disability.

- Essential
- Important
- Don't know/depends
- Unimportant
- Should not be included

* 216. If the first aider calls a mental health crisis team, they should find out if they will be attending with the police, and let the person know if this is the case.

- Essential
- Important
- Don't know/depends
- Unimportant
- Should not be included

* 217. If police attend, the first aider should reassure the person that they are not in trouble.

- Essential
- Important
- Don't know/depends
- Unimportant
- Should not be included

* 218. If police attend, the first aider should tell them that the person has an intellectual disability and appears to be experiencing a mental health crisis and requires medical help.

- Essential
- Important
- Don't know/depends
- Unimportant
- Should not be included

* 219. The first aider should try to meet emergency staff on arrival to remind them that the person has an intellectual disability before they approach the person.

- Essential
- Important
- Don't know/depends
- Unimportant
- Should not be included

220. Please provide any additional items or comments related to this section.

Development of guidelines for considerations when providing mental health first aid to a person with an intellectual disability

CRISIS SITUATION cont.

This section contains statements about what the first aider needs to know and do if they are concerned the person is suicidal.

Please rate how important (from ‘essential’ to ‘should not be included’) you think it is that each statement be included in the guidelines.

Please keep our definitions in mind when responding to this section. You can access the definitions here.

Suicide

* 221. If the person has a carer or support person with them, the first aider should share their concerns with them and ask if they are also concerned the person may be at risk of suicide.

- ◯ Essential
- ◯ Important
- ◯ Don't know/depends
- ◯ Unimportant
- ◯ Should not be included

* 222. The first aider should know that the person may not understand the term "suicide" and may need to use words such as "kill yourself" or "make yourself die" instead.

- ◯ Essential
- ◯ Important
- ◯ Don't know/depends
- ◯ Unimportant
- ◯ Should not be included

223. Please provide any additional items or comments related to this section.

Development of guidelines for considerations when providing mental health first aid to a person with an intellectual disability

IF THE PERSON NEEDS TO BE TAKEN TO HOSPITAL

This section contains statements about what the first aider needs to know and do if the person needs to be taken to hospital.

Mental health first aid is the help offered to a person developing a mental health problem, experiencing a worsening of an existing mental health problem, or in a mental health crisis. The first aid is given until appropriate professional help is received or until the crisis resolves.

Therefore, if the person needs to go to hospital, the role of the mental health first aider ceases once the person receives appropriate professional help (e.g. via GP/family doctor, health professional etc). If the first aider is also a carer or support person or disability worker, they may continue to provide ongoing support to the person once they have been seen by a professional. When this occurs, they are no longer acting in their role as a first aider but are providing ongoing support in their usual role. For this reason, this section does not cover ongoing support once the person has received appropriate professional help.

Please rate how important (from ‘essential’ to ‘should not be included’) you think it is that each statement be included in the guidelines.

Please keep our definitions in mind when responding to this section. You can access the definitions here.

If the person needs to be taken to hospital

* 224. If the person needs to be taken to hospital, the first aider should be aware that hospital can be particularly stressful for a person with an intellectual disability, due to factors such as noise, unfamiliar faces, or worries about their health or being in trouble, or having difficulty understanding what is happening around them.

- Essential
- Important
- Don't know/depends
- Unimportant
- Should not be included

* 225. If the person needs to be taken to hospital, the first aider should try to explain to the person what is likely to happen.

- Essential
- Important
- Don't know/depends
- Unimportant
- Should not be included

* 226. If the person needs to be taken to hospital, the first aider should contact the hospital in advance to make sure they are aware the person has an intellectual disability and if any reasonable adjustments may be needed, with the person's permission.

- Essential
- Important
- Don't know/depends
- Unimportant
- Should not be included

* 227. If the person needs to be transported to hospital, the first aider should try to ensure the person has a support person or carer accompanying them where possible.

- Essential
- Important
- Don't know/depends
- Unimportant
- Should not be included

* 228. If the first aider is aware that the person has previously had a negative experience with the emergency department, they should inform the emergency responders of this.

- Essential
- Important
- Don't know/depends
- Unimportant
- Should not be included

* 229. If the first aider accompanies the person to hospital, they should provide support until they see a health professional or a family member or carer arrives, e.g. requesting a quiet area to wait, ensuring the person's basic needs are met.

- Essential
- Important
- Don't know/depends
- Unimportant
- Should not be included

230. Please provide any additional items or comments related to this section.

Development of guidelines for considerations when providing mental health first aid to a person with an intellectual disability

Thank you!

Thank you for sharing your expertise and time with us.

If anything in this survey has caused you distress and you would like to talk with someone about it you can contact the appropriate crisis help line below:

Australia: Lifeline on 13 11 14

Canada: National Suicide prevention Lifeline on 1800 273 TALK (8255)

Denmark: Suicide hotline 70 201 201

Finland: SOS Crisis Centre 010 195 202

France: Suicide Écoute 01 45 39 40 00

The Netherlands: Suicide hotline 0900 0113

New Zealand: Lifeline Aotearoa on 0800 543 354

Republic of Ireland: Samaritans on 116 123

Sweden: Suicide hotline 020 22 00 60

Switzerland: PARSPAS 027 321 21 21

UK: Samaritans on 116 123

USA: National Suicide prevention Lifeline on 1800 273 TALK (8255)

Round 2: Development of guidelines for considerations when providing mental health first aid to a person with an intellectual disability

Information

How this questionnaire was developed
The statements in this questionnaire were derived from the results of the first survey. This survey includes statements from the Round 1 survey that need to be rerated (marked with the word "Rerate") and statements that were developed from participant Round 1 comments (marked with the word "New").

Some of the statements may seem contradictory or controversial; however, they have been included them because they reflect the wide range of people's beliefs about the best ways to provide mental health first aid to a person who has an intellectual disability and is experiencing mental health problems. ***It is important to note that the researchers have not made judgements about the statements in the survey and do not necessarily agree or disagree with them.*** The purpose of the survey is to obtain a consensus view about each of the statements from the expert panel.

Definitions used in this survey

Intellectual disability: is characterised by:
Global deficiencies in intellectual and adaptive functioning, where the person may not reach expected developmental milestones. The deficiencies in the intellectual functions and adaptability begin during childhood or adolescence.

Mental health first aid: is the help offered to a person developing a mental health problem, experiencing a worsening of an existing mental health problem, or in a mental health crisis. The first aid is given until appropriate professional help is received or until the crisis resolves.

The person: a person who has an intellectual disability, who the mental health first aider (first aider) is concerned may be experiencing a mental health problem.

The first aider: a family member, friend, concerned community member or disability worker without specialist mental health qualifications who provides initial help to a person who has an intellectual disability and is experiencing mental health problems.

Carer or support person: refers to people who provide paid or unpaid personal care, support and assistance to a person with an intellectual disability. It can include a spouse, de facto partner, parent, other relative or guardian, or disability support worker.

Disability worker: a person paid to support someone with an intellectual disability. They may or may not be formally trained.

GP/Family doctor: a medical doctor based in the community who treats patients with minor or chronic illnesses and refers those with serious conditions to a specialist or hospital.

Professional/health professional: a broad range of health professionals through which a person may seek help for mental health problems. This could include a mental health professional, GP/family doctor, or another health professional, e.g. allied health professional, hospital emergency staff.

Mental health professional: a health professional who is qualified to treat people who are experiencing mental health problems, e.g. a psychologist, mental health nurse, psychiatrist, or social worker or occupational therapist with specialist mental health training.

Emergency services: services that respond to and deal with emergencies when they occur, e.g. emergency medical services (ambulance) or law enforcement (the police).

Mental health crisis service: services that respond to and provide immediate help during a mental health crisis and are responsible for assessing the care required by the person. Psychiatric nurses, social workers, psychiatrists and psychologists may work for a mental health crisis service.

Mental health crisis/crisis: a situation in which:
- The person may harm themselves, e.g. by attempting suicide, by using substances to become intoxicated, by engaging in non-suicidal self-injury, or as a result of extreme weight loss
- The person experiences extreme distress, e.g. a panic attack, a traumatic event or a severe psychotic state
- The person's behaviour is very disturbing to others, e.g. they become aggressive or lose touch with reality.

Abuse: mistreatment that occurs between people (interpersonal trauma), e.g. emotional, physical or sexual abuse including family violence, child abuse, and elder abuse.

Trauma: an emotional response to a powerful and distressing experience. Immediate trauma responses can include shock and denial. Longer term reactions include unpredictable emotions, flashbacks, strained relationships and physical symptoms like headaches or nausea. Powerful and distressing experiences are usually life threatening or pose a significant threat to a person's physical or psychological wellbeing.

Some common examples events that have the potential to cause trauma include interpersonal violence (including family violence, child abuse, elder abuse, physical or sexual assault, mugging or robbery), accidents (such as traffic or workplace accidents), and witnessing something terrible happen. Mass traumatic events include war, terrorist attacks, mass shootings, and severe weather events (flood, earthquake, hurricane, tsunami, forest and bush fire).

Indirect exposure can also cause trauma, for example witnessing others experience a potentially traumatic event, learning that a potentially traumatic event occurred to someone you know, or repeated or extreme exposure to details of a potentially traumatic event.

Non-suicidal self-injury: self injury that is not intended to result in death. It does not include behaviours that may cause unintentional injury to the person (e.g. stimming behaviours).

Self-injury/self-injurious behaviour: behaviour that has the potential to cause physical injury to oneself and can be for a range of reasons, including to communicate a need such as hunger, to manage distress (NSSI), or as a result of stimming behaviours.

Self-stimulatory behaviour (stimming): the repetition of physical movements, sounds, or repetitive movement of objects common in people with an intellectual disability. Stimming behaviours include hand flapping, rocking, excessive or hard blinking, pacing, head banging, hitting the head, pulling the hair, repeating noises or words, snapping fingers, and spinning objects. While stimming can be an outlet for the person - e.g. to gain/reduce sensory input if the person has sensory sensitivities, some stimming behaviours can cause unintentional injury to the person (e.g. hitting the head or pulling the hair).

'*Challenging behaviour*': Behaviour of such intensity, frequency or duration that the physical safety of the person or others is placed in serious jeopardy, or behaviour which is likely to seriously limit or deny access to the use of ordinary community facilities. It can include a range of behaviours, for example physical aggression towards objects or people, self-injury, sexually inappropriate behaviour, offending behaviour (such as arson or stealing), mannerisms or ritual.

Round 2: Development of guidelines for considerations when providing mental health first aid to a person with an intellectual disability

Instructions

Instructions
There are already guidelines to help people who may be experiencing a range of mental health problems (go to our website to access these guidelines). We do not wish to replicate existing MHFA guidelines. Rather, the aim of this project is to develop guidelines on how to tailor mental health first aid to help a person who has an intellectual disability and is experiencing mental health problems. The guidelines that results from this project will be used in conjunction with the existing mental health first aid guidelines.

Please complete the questionnaire by rating each statement according to how important you believe it is for inclusion in the guidelines for considerations when providing mental health first aid to a who has an intellectual disability and is experiencing mental health problems. Please keep in mind that the guidelines will be used by family members, friends, concerned community members or disability workers without specialist mental health qualifications. The guidelines will not be for mental health professionals who are looking for guidance on working with patients with intellectual disabilities.

The statements need to be rated according to their importance for someone ***WITHOUT clinical background.***

This questionnaire should take approximately 30 minutes to complete. You can complete the survey in two or more sittings. Your answers are saved when you click 'Next' at the bottom of a page. This marks your page and you can begin again at a later date on the next page. **Please be aware that once you have logged on and started responding you must complete the questionnaire on the same computer.**

The next phase of the research involves completing one more short surveys.

Overview of the questionnaire
Section 1: Knowing about intellectual disability and mental health problems
Section 2: Recognising and acknowledging a person may be experiencing mental health problems
Section 3: Raising concerns with the person
Section 4: Communicating with the person
Section 5: Respect and rights
Section 6: Being supportive
Section 7: Anxiety
Section 8: Self-injury
Section 9: Substance use
Section 10: Eating disorders
Section 11: Abuse
Section 12: Other supports
Section 13: Difficulties the first aider may encounter
Section 14: Professional help
Section 15: Crisis situation
Section 16: If the person needs to be taken to hospital

For more information
You received a Plain Language Statement when you expressed interest in this project. Please refer to this for more details about this study.

Round 2: Development of guidelines for considerations when providing mental health first aid to a person with an intellectual disability

Information about you

* 1. What is your name? (This allows us to determine who has completed the Round 1 survey and is therefore eligible to participate in Round 2. Your name will be deleted from your data when the project is complete).

Round 2: Development of guidelines for considerations when providing mental health first aid to a person with an intellectual disability

KNOWING ABOUT INTELLECTUAL DISABILITY AND MENTAL HEALTH PROBLEMS

This section contains statements about what the first aider needs to know about intellectual disability and mental health problems

Please rate how important (from ‘essential’ to ‘should not be included’) you think it is that each statement be included in the guidelines.

Please keep our definitions in mind when responding to this section. You can access the definitions here.

Knowing about intellectual disability and mental health problems

* 2. The first aider should not make any assumptions as to the cognitive ability of the person. (New)

- Essential
- Important
- Don't know/depends
- Unimportant
- Should not be included

Round 2: Development of guidelines for considerations when providing mental health first aid to a person with an intellectual disability

RECOGNISING AND ACKNOWLEDGING A PERSON MAY BE EXPERIENCING MENTAL HEALTH PROBLEMS

This section contains statements about what the first aider needs to know about recognising and acknowledging that a person with an intellectual disability may be experiencing mental health problems.

Please rate how important (from ‘essential’ to ‘should not be included’) you think it is that each statement be included in the guidelines.

Please keep our definitions in mind when responding to this section. You can access the definitions here.

Recognising and acknowledging a person with an intellectual disability may be experiencing mental health problems.

PLEASE NOTE:
The following three statements apply **only** to situations in which the first aider is a **paid disability worker.**
Please rate how important (from 'essential' to 'should not be included') you think it is that each statement be included in the guidelines when the first aider is a **paid disability worker.**

* 3. The first aider should discuss the changes they have noticed in the person with a case manager or employer. (Rerate)

- ◯ Essential
- ◯ Important
- ◯ Don't know/depends
- ◯ Unimportant
- ◯ Should not be included

* 4. If the person is not experiencing a mental health crisis, the first aider should talk to an appropriate work colleague (e.g. someone who has spent time with the person) to see if anyone else has noticed similar changes in the person's behaviour or mood. (New)

- ◯ Essential
- ◯ Important
- ◯ Don't know/depends
- ◯ Unimportant
- ◯ Should not be included

* 5. If the person is not experiencing a mental health crisis, the first aider should discuss the changes they have noticed with **an appropriate work colleague** (e.g. supervisor, manager, case manager) and **with appropriate consent** (e.g. with the person, their guardian, or dependant on the complexity of the situation). (New)

- ◯ Essential
- ◯ Important
- ◯ Don't know/depends
- ◯ Unimportant
- ◯ Should not be included

* 6. If the person does not consent to the first aider discussing the person's mental health with an appropriate work colleague (e.g. supervisor, manager, case manager), they should seek advice without identifying the person. (New)

- Essential
- Important
- Don't know/depends
- Unimportant
- Should not be included

* 7. If the first aider needs to record information about any changes in the person's behaviour or mood, they should do this in a factual way rather than giving an opinion, and only with the person's permission. (Rerate)

- Essential
- Important
- Don't know/depends
- Unimportant
- Should not be included

* 8. If the first aider is **required by their employer** to record information about any changes in the person's behaviour or mood, they should do this in a factual way rather than giving an opinion. (New)

- Essential
- Important
- Don't know/depends
- Unimportant
- Should not be included

Round 2: Development of guidelines for considerations when providing mental health first aid to a person with an intellectual disability

RAISING CONCERNS WITH THE PERSON

This section contains statements about what the first aider needs to know about raising their concerns with the person.

Please rate how important (from ‘essential’ to ‘should not be included’) you think it is that each statement be included in the guidelines.

Please keep our definitions in mind when responding to this section. You can access the definitions here.

Raising concerns with the person

* 9. The first aider should not assume they know the best way of helping the person and should be guided by any instruction the person, their carer or their legal guardian may give. (New)

- Essential
- Important
- Don't know/depends
- Unimportant
- Should not be included

* 10. If the first aider offers to help the person and the person does not want their help, the first aider should respect this, unless there is a risk of harm to the person or others. (New)

- Essential
- Important
- Don't know/depends
- Unimportant
- Should not be included

* 11. When providing mental health first aid, the first aider should consider the person's capacity* to respond and engage with any suggestions or advice, e.g. support services, self-help strategies or information. (New)
**Capacity includes the person's abilities, their environment and their support.*

- Essential
- Important
- Don't know/depends
- Unimportant
- Should not be included

Round 2: Development of guidelines for considerations when providing mental health first aid to a person with an intellectual disability

COMMUNICATING WITH THE PERSON

This section contains statements about what the first aider needs to know and do when communicating with the person about their symptoms.

Please rate how important (from ‘essential’ to ‘should not be included’) you think it is that each statement be included in the guidelines.

Please keep our definitions in mind when responding to this section. You can access the definitions here.

There are four parts to this section:
1. Communicating with the person
2. Comprehension
3. Questions
4. Engaging others in the conversation

Communicating with the person

* 12. The first aider should use simple, short statements or questions. (Rerate)

- Essential
- Important
- Don't know/depends
- Unimportant
- Should not be included

* 13. The first aider should use descriptive rather than technical language. (Rerate)

- Essential
- Important
- Don't know/depends
- Unimportant
- Should not be included

* 14. The first aider should not use metaphors, sarcasm or idioms (e.g. "under the weather"), as the person may misunderstand these. (Rerate)

- Essential
- Important
- Don't know/depends
- Unimportant
- Should not be included

* 15. The first aider should seek advice from someone that knows the person well about the best way to communicate with them. (New)

- Essential
- Important
- Don't know/depends
- Unimportant
- Should not be included

* 16. The first aider should determine if the person has a document that explains their communication preferences, e.g. communication passport. (New)

- ◯ Essential
- ◯ Important
- ◯ Don't know/depends
- ◯ Unimportant
- ◯ Should not be included

* 17. The first aider should be flexible in their communication style with the person. (New)

- ◯ Essential
- ◯ Important
- ◯ Don't know/depends
- ◯ Unimportant
- ◯ Should not be included

* 18. As people with an intellectual disability may not indicate when they do not understand something, the first aider should tell the person that it is ok to say if they don't understand, e.g. by saying "I need to make sure I explain it properly. Please let me know if I am not clear enough." (New)

- ◯ Essential
- ◯ Important
- ◯ Don't know/depends
- ◯ Unimportant
- ◯ Should not be included

* 19. If the person is distressed, the first aider should try to make the environment less stressful for them (e.g. turning off the TV or asking people to be quiet) as the person may be responding to overstimulation. (Rerate)

- ◯ Essential
- ◯ Important
- ◯ Don't know/depends
- ◯ Unimportant
- ◯ Should not be included

* 20. If the person is distressed, the first aider should **try to identify the cause** of the distress and make changes where appropriate, e.g. turning off the TV, asking people to be quiet, moving to another location. (New)

- Essential
- Important
- Don't know/depends
- Unimportant
- Should not be included

* 21. If the person is using stimming behaviours or other distractions to manage their emotions during the conversation, the first aider should not attempt to stop them, unless they are harming themselves or others. (New)

- Essential
- Important
- Don't know/depends
- Unimportant
- Should not be included

* 22. The first aider should be aware that some people with an intellectual disability may respond with an unexpected physical response, e.g. hugging or kissing new people. (Rerate)

- Essential
- Important
- Don't know/depends
- Unimportant
- Should not be included

Round 2: Development of guidelines for considerations when providing mental health first aid to a person with an intellectual disability

COMMUNICATING WITH THE PERSON cont.

Please rate how important (from ‘essential’ to ‘should not be included’) you think it is that each statement be included in the guidelines.

Please keep our definitions in mind when responding to this section. You can access the definitions here.

Comprehension

* 23. The first aider should reinforce important messages to increase the likelihood the person will understand and remember. (Rerate)

- Essential
- Important
- Don't know/depends
- Unimportant
- Should not be included

* 24. If the person is not at risk of harm, the first aider should find out if there is a time of day/day of week where the person is more able to understand and communicate information. (New)

- Essential
- Important
- Don't know/depends
- Unimportant
- Should not be included

* 25. The first aider should know that frequent breaks or multiple short sessions might be required to enhance comprehension and communication. (New)

- Essential
- Important
- Don't know/depends
- Unimportant
- Should not be included

* 26. If the first aider thinks the person has lost track of the conversation, they should ask the person to share back their understanding of what the first aider has said. (New)

- Essential
- Important
- Don't know/depends
- Unimportant
- Should not be included

* 27. The first aider should try to help the person identify and label what they are feeling, e.g. frustrated. (Rerate)

- ◯ Essential
- ◯ Important
- ◯ Don't know/depends
- ◯ Unimportant
- ◯ Should not be included

Round 2: Development of guidelines for considerations when providing mental health first aid to a person with an intellectual disability

COMMUNICATING WITH THE PERSON cont.

Please rate how important (from ‘essential’ to ‘should not be included’) you think it is that each statement be included in the guidelines.

Please keep our definitions in mind when responding to this section. You can access the definitions here.

Questions

* 28. If the first aider asks the person a question, they should wait for a response before asking a second question. (Rerate)

- ◯ Essential
- ◯ Important
- ◯ Don't know/depends
- ◯ Unimportant
- ◯ Should not be included

* 29. The first aider should start by asking the person questions they know the person can answer, as this can help to reduce the person's anxiety and build confidence. (Rerate)

- ◯ Essential
- ◯ Important
- ◯ Don't know/depends
- ◯ Unimportant
- ◯ Should not be included

Round 2: Development of guidelines for considerations when providing mental health first aid to a person with an intellectual disability

COMMUNICATING WITH THE PERSON cont.

Please rate how important (from ‘essential’ to ‘should not be included’) you think it is that each statement be included in the guidelines.

Please keep our definitions in mind when responding to this section. You can access the definitions here.

Engaging others in the conversation

* 30. The first aider should direct all communication to the person, even if the person has chosen to have someone else present. (New)

- Essential
- Important
- Don't know/depends
- Unimportant
- Should not be included

* 31. If a legal guardian or other person is present and the first aider wants to ask them a question, they should check with the person if this is okay before doing so. (New)

- Essential
- Important
- Don't know/depends
- Unimportant
- Should not be included

* 32. Even if a guardian or other person is present and needs to be consulted, the first aider should ensure the person with intellectual disability is the centre of the conversation at all times. (New)

- Essential
- Important
- Don't know/depends
- Unimportant
- Should not be included

* 33. If the first aider believes that the person is feeling pressured or less able to speak openly because someone else is present, they should meet with the person alone, if this is possible and the person wants to. (New)

- Essential
- Important
- Don't know/depends
- Unimportant
- Should not be included

Round 2: Development of guidelines for considerations when providing mental health first aid to a person with an intellectual disability

RESPECT AND RIGHTS

This section contains statements about what the first aider needs to know about respecting the person and their rights.

Please rate how important (from ‘essential’ to ‘should not be included’) you think it is that each statement be included in the guidelines.

Please keep our definitions in mind when responding to this section. You can access the definitions here.

Respecting the person and their rights

* 34. The first aider should try to find out if the person has a legal guardian or conservator appointed. (Rerate)

- Essential
- Important
- Don't know/depends
- Unimportant
- Should not be included

* 35. If the first aider needs to ask someone else for information, they should get **the person's permission** before doing so. (Rerate)

- Essential
- Important
- Don't know/depends
- Unimportant
- Should not be included

* 36. If the first aider needs to ask someone else for information, they should get **appropriate consent (i.e. from the person or their legal guardian)** before doing so. (New)

- Essential
- Important
- Don't know/depends
- Unimportant
- Should not be included

* 37. If the first aider needs to share information about the person with anyone else, they should get **the person's permission** before doing so. (Rerate)

- Essential
- Important
- Don't know/depends
- Unimportant
- Should not be included

* 38. If the first aider needs to share information about the person with anyone else, they should **get appropriate consent**, i.e. from the person or their legal guardian. (New)

- Essential
- Important
- Don't know/depends
- Unimportant
- Should not be included

* 39. The first aider should not involve another person in the first aid without the person's permission, unless there is risk of harm to the person or others. (Rerate)

- Essential
- Important
- Don't know/depends
- Unimportant
- Should not be included

* 40. If after talking with the person, the first aider thinks some of the information should be conveyed to a legal guardian, they should do so with the person present. (New)

- Essential
- Important
- Don't know/depends
- Unimportant
- Should not be included

Round 2: Development of guidelines for considerations when providing mental health first aid to a person with an intellectual disability

BEING SUPPORTIVE

This section contains statements about what the first aider needs to know about being supportive to a person who may be experiencing mental health problems.

Please rate how important (from ‘essential’ to ‘should not be included’) you think it is that each statement be included in the guidelines.

Please keep our definitions in mind when responding to this section. You can access the definitions here.

Being supportive

* 41. The first aider should ask the person if they would like any practical assistance with tasks while being careful not to take over or encourage dependency. (Rerate)

- Essential
- Important
- Don't know/depends
- Unimportant
- Should not be included

* 42. If the first aider observes that the person may need some practical assistance, they should try to facilitate this support, i.e. provide the support themselves or find an appropriate person. (New)

- Essential
- Important
- Don't know/depends
- Unimportant
- Should not be included

* 43. The first aider should ask the person if they have felt this way before, and if so, what they have done in the past that has been helpful. (New)

- Essential
- Important
- Don't know/depends
- Unimportant
- Should not be included

* 44. The first aider should ask the person if they need support to use coping strategies for their mental health problems. (Rerate)

- Essential
- Important
- Don't know/depends
- Unimportant
- Should not be included

* 45. Before offering specific supports, the first aider should check what supports are available to the person. (New)

- Essential
- Important
- Don't know/depends
- Unimportant
- Should not be included

* 46. The first aider should ask the person if they would like some information about mental health problems and in what form they would like the information. (Rerate)

- Essential
- Important
- Don't know/depends
- Unimportant
- Should not be included

* 47. If the person needs additional support, but family and friends are under stress or 'burnt out', the first aider should assist the person to find alternate supports. (Rerate)

- Essential
- Important
- Don't know/depends
- Unimportant
- Should not be included

Round 2: Development of guidelines for considerations when providing mental health first aid to a person with an intellectual disability

ANXIETY

This section contains statements about what the first aider needs to know if they are concerned the person may be experiencing anxiety.

Please rate how important (from ‘essential’ to ‘should not be included’) you think it is that each statement be included in the guidelines.

Please keep our definitions in mind when responding to this section. You can access the definitions here.

Anxiety

* 48. The first aider should know that special interests and routines, although seemingly 'obsessive' may help the person cope with stress and they should not be encouraged to change these. (New)

- Essential
- Important
- Don't know/depends
- Unimportant
- Should not be included

Round 2: Development of guidelines for considerations when providing mental health first aid to a person with an intellectual disability

SELF-INJURY

This section contains statements about what the first aider needs to know if they are concerned the person may be injuring themselves.

Please rate how important (from ‘essential’ to ‘should not be included’) you think it is that each statement be included in the guidelines.

Please keep our definitions in mind when responding to this section. You can access the definitions here.

Self-injury

* 49. If the person lives with their family or in a residential care setting and is self-injuring, the first aider should seek assistance from family or staff. (New)

- Essential
- Important
- Don't know/depends
- Unimportant
- Should not be included

* 50. If the person is self-injuring, the first aider should refer to their behaviour support plan, if there is one in place. (New)

- Essential
- Important
- Don't know/depends
- Unimportant
- Should not be included

* 51. If the person is self-injurying, the first aider should try to minimise the person's risk of immediate harm, e.g. putting a pillow between the person's head and a wall. (Rerate)

- Essential
- Important
- Don't know/depends
- Unimportant
- Should not be included

* 52. The first aider should know that physical restraint should only be considered as a last resort when there is risk of serious harm. (New)

- Essential
- Important
- Don't know/depends
- Unimportant
- Should not be included

SUBSTANCE USE

This section contains statements about what the first aider needs to know if they are concerned the person may be misusing substances.

Please rate how important (from ‘essential’ to ‘should not be included’) you think it is that each statement be included in the guidelines.

Please keep our definitions in mind when responding to this section. You can access the definitions here.

Substance use

* 53. The first aider should ask the person about their substance use (e.g. what, how much, for how long), rather than using clinical terms such as 'substance misuse' or 'substance abuse'. (Rerate)

- Essential
- Important
- Don't know/depends
- Unimportant
- Should not be included

* 54. The first aider should know that the person may be susceptible to peer pressure to use substances. (Rerate)

- Essential
- Important
- Don't know/depends
- Unimportant
- Should not be included

* 55. The first aider should not dismiss the person's substance use as understandable in their circumstances, e.g. people may think "I would drink too if I were them." (Rerate)

- Essential
- Important
- Don't know/depends
- Unimportant
- Should not be included

EATING DISORDERS

This section contains statements about what the first aider needs to know if they are concerned the person may have an eating disorder.

Please rate how important (from ‘essential’ to ‘should not be included’) you think it is that each statement be included in the guidelines.

Please keep our definitions in mind when responding to this section. You can access the definitions here.

Eating disorders

* 56. The first aider should know that there are certain conditions and medications that may affect the person's eating habits. (New)

- Essential
- Important
- Don't know/depends
- Unimportant
- Should not be included

Round 2: Development of guidelines for considerations when providing mental health first aid to a person with an intellectual disability

ABUSE

This section contains statements about what the first aider needs to know if they are concerned the person may be experiencing abuse.

Please rate how important (from ‘essential’ to ‘should not be included’) you think it is that each statement be included in the guidelines.

Please keep our definitions in mind when responding to this section. You can access the definitions here.

Abuse

* 57. If the first aider has reason to believe the person may be experiencing abuse, the first aider should seek advice from an appropriate service as conversations with the person may contaminate evidence that could be critical to future legal proceedings. (New)

- Essential
- Important
- Don't know/depends
- Unimportant
- Should not be included

* 58. If the first aider has reason to believe the person may be experiencing abuse, they should encourage the person to report this to an appropriate service, e.g. the police, disability abuse and neglect hotline, crisis support service. (Rerate)

- Essential
- Important
- Don't know/depends
- Unimportant
- Should not be included

* 59. If the first aider assists the person to report abuse, they should link the person in with supports that will help them through the process of reporting, e.g. advocates, housing support. (New)

- Essential
- Important
- Don't know/depends
- Unimportant
- Should not be included

* 60. The first aider should try to talk to the person without the presence of others who may influence their ability to speak freely. (Rerate)

- Essential
- Important
- Don't know/depends
- Unimportant
- Should not be included

* 61. If the first aider has reason to believe the person may be experiencing abuse and is in immediate danger, they should call the police. (Rerate)

- Essential
- Important
- Don't know/depends
- Unimportant
- Should not be included

* 62. If the first aider has reason to believe the person may be experiencing abuse and is in immediate danger, they should **encourage the person** to call the police. (Rerate)

- Essential
- Important
- Don't know/depends
- Unimportant
- Should not be included

* 63. If the first aider has reason to believe the person may be experiencing abuse and is in immediate danger **of physical harm**, and the person is does not want to call the police, the first aider should call on their behalf. (New)

- Essential
- Important
- Don't know/depends
- Unimportant
- Should not be included

Round 2: Development of guidelines for considerations when providing mental health first aid to a person with an intellectual disability

OTHER SUPPORTS

This section contains statements about what the first aider needs to know about encouraging other supports.

Please rate how important (from ‘essential’ to ‘should not be included’) you think it is that each statement be included in the guidelines.

Please keep our definitions in mind when responding to this section. You can access the definitions here.

Other supports

* 64. The first aider should ask the person if they have used self-help strategies in the past that they found helpful, and if so, support them to use these if needed. (Rerate)

- Essential
- Important
- Don't know/depends
- Unimportant
- Should not be included

* 65. If the person has used self-help strategies in the past that they found helpful, the first aider should encourage them to ask others to support them in their use. (New)

- Essential
- Important
- Don't know/depends
- Unimportant
- Should not be included

* 66. If the person needs additional support, but family and friends are under stress or 'burnt out', the first aider should assist the person to find this support. (New)

- Essential
- Important
- Don't know/depends
- Unimportant
- Should not be included

* 67. If the person wants to use self-help strategies but needs support to do so, the first aider should suggest a disability-specific service (where available) that offers specialised education programs to assist them to learn these skills. (Rerate)

- Essential
- Important
- Don't know/depends
- Unimportant
- Should not be included

* 68. If the person wants to **learn to use** self-help strategies but needs support to do so, the first aider should suggest **a support service** (where available) that offers specialised education programs to assist them to learn these skills. (New)

- Essential
- Important
- Don't know/depends
- Unimportant
- Should not be included

Round 2: Development of guidelines for considerations when providing mental health first aid to a person with an intellectual disability

DIFFICULTIES THE FIRST AIDER MAY ENCOUNTER

This section contains statements about what a first aider should know and do regarding difficulties they may encounter.

Please rate how important (from ‘essential’ to ‘should not be included’) you think it is that each statement be included in the guidelines.

Please keep our definitions in mind when responding to this section. You can access the definitions here.

There are three parts to this section:
1. General
2. Aggressive behaviours
3. Sexually inappropriate behaviours

General

* 69. If the person is exhibiting behaviours that the first aider finds challenging, the first aider should know that trying to stop or control the behaviour without addressing how the person is feeling is not likely to be successful. (Rerate)

- Essential
- Important
- Don't know/depends
- Unimportant
- Should not be included

* 70. If the person is exhibiting behaviours that the first aider finds challenging, the first aider should try to redirect the person away from those behaviours, e.g. "I can see you're really upset, do you want to go for a walk?" (New)

- Essential
- Important
- Don't know/depends
- Unimportant
- Should not be included

* 71. If the person is exhibiting behaviours that the first aider finds challenging, the first aider should try to make changes in the environment (e.g. turning off the TV or asking people to be quiet) as the person may be responding to overstimulation. (New)

- Essential
- Important
- Don't know/depends
- Unimportant
- Should not be included

* 72. The first aider should encourage the person to express their feelings through verbal communication or communication tools instead of through the behaviour that the first aider is finding challenging. (Rerate)

- Essential
- Important
- Don't know/depends
- Unimportant
- Should not be included

Round 2: Development of guidelines for considerations when providing mental health first aid to a person with an intellectual disability

DIFFICULTIES THE FIRST AIDER MAY ENCOUNTER cont.

Please rate how important (from ‘essential’ to ‘should not be included’) you think it is that each statement be included in the guidelines.

Please keep our definitions in mind when responding to this section. You can access the definitions here.

Aggressive behaviours

* 73. If the person is behaving aggressively, the first aider should try to enlist the help of someone who knows the person better. (New)

- Essential
- Important
- Don't know/depends
- Unimportant
- Should not be included

* 74. **If the first aider is a disability worker**, they should be familiar with the person's behaviour support plan, if there is one in place. (New)

- Essential
- Important
- Don't know/depends
- Unimportant
- Should not be included

Round 2: Development of guidelines for considerations when providing mental health first aid to a person with an intellectual disability

DIFFICULTIES THE FIRST AIDER MAY ENCOUNTER cont.

Please rate how important (from 'essential' to 'should not be included') you think it is that each statement be included in the guidelines.

Please keep our definitions in mind when responding to this section. You can access the definitions here.

Sexually inappropriate behaviours

Sexually inappropriate behaviour is sexual behaviour that negatively impacts on others or is outside of legislation. It is characterised by a range of activities that can be persistent, frequent, harmful or between unwilling or unequal participants (e.g. in age, size, ability).

* 75. If the person is exhibiting sexually inappropriate behaviour, the first aider should redirect them, e.g. to another activity or to a private area. (New)

- Essential
- Important
- Don't know/depends
- Unimportant
- Should not be included

* 76. If the person is exhibiting sexually inappropriate behaviour, the first aider should check with the person's carer or professional to see if any strategies have been put in place. (New)

- Essential
- Important
- Don't know/depends
- Unimportant
- Should not be included

* 77. If the person is exhibiting sexually inappropriate behaviour, the first aider should tell their carer or other support person. (New)

- Essential
- Important
- Don't know/depends
- Unimportant
- Should not be included

Round 2: Development of guidelines for considerations when providing mental health first aid to a person with an intellectual disability

PROFESSIONAL HELP

This section contains statements about what the first aider needs to know and do when encouraging the person to seek professional help.

Please rate how important (from 'essential' to 'should not be included') you think it is that each statement be included in the guidelines.

Please keep our definitions in mind when responding to this section. You can access the definitions here.

There are three parts to this section:

1. Professional help
2. Supporting the person to attend a professional appointment
3. If the person has an appointment with a professional

Professional help

* 78. The first aider should respect the person's right to make their own decisions regarding seeking professional help. (Rerate)

- Essential
- Important
- Don't know/depends
- Unimportant
- Should not be included

* 79. If the person has a legal guardian, the first aider should involve them with regards to seeking professional help. (New)

- Essential
- Important
- Don't know/depends
- Unimportant
- Should not be included

* 80. The first aider should provide the person with information that may help them to make a decision about seeking professional help, e.g. what is involved in the various options and the benefits of each option. (Rerate)

- Essential
- Important
- Don't know/depends
- Unimportant
- Should not be included

PROFESSIONAL HELP cont.

This section contains statements about what the first aider needs to know and do when supporting the person to attend a professional appointment.

Please rate how important (from ‘essential’ to ‘should not be included’) you think it is that each statement be included in the guidelines.

Please keep our definitions in mind when responding to this section. You can access the definitions here.

Supporting the person to attend an appointment

* 81. The first aider should offer the person the support they require to make an appointment with a professional. (Rerate)

- Essential
- Important
- Don't know/depends
- Unimportant
- Should not be included

* 82. If appropriate to the relationship, the first aider should support the person to make an appointment with a health professional. (Rerate)

- Essential
- Important
- Don't know/depends
- Unimportant
- Should not be included

* 83. If challenges arise that prevent the person from receiving appropriate professional help, the first aider should try to find an advocate who can support the person to access appropriate help. (Rerate)

- Essential
- Important
- Don't know/depends
- Unimportant
- Should not be included

Round 2: Development of guidelines for considerations when providing mental health first aid to a person with an intellectual disability

PROFESSIONAL HELP cont.

This section contains statements about what the first aider needs to know and do if the person has an appointment with a professional.

Please rate how important (from 'essential' to 'should not be included') you think it is that each statement be included in the guidelines.

Please keep our definitions in mind when responding to this section. You can access the definitions here.

If the person has an appointment with a professional

* 84. The first aider should explain to the person what they may expect in their appointment. (Rerate)

- Essential
- Important
- Don't know/depends
- Unimportant
- Should not be included

* 85. The first aider should help the person to anticipate the upcoming appointment by making sure they know the time and place, and who they will see. (Rerate)

- Essential
- Important
- Don't know/depends
- Unimportant
- Should not be included

* 86. If the person has a book or document explaining their communication needs, the first aider should encourage the person to take this to their appointment. (New)

- Essential
- Important
- Don't know/depends
- Unimportant
- Should not be included

* 87. If the first aider accompanies the person to an appointment, they should assist the professional to communicate and interact with the person, with the person's permission. (Rerate)

- Essential
- Important
- Don't know/depends
- Unimportant
- Should not be included

Round 2: Development of guidelines for considerations when providing mental health first aid to a person with an intellectual disability

CRISIS SITUATION

This section contains statements about what the first aider needs to know and do if the person is in crisis.

Please rate how important (from ‘essential’ to ‘should not be included’) you think it is that each statement be included in the guidelines.

Please keep our definitions in mind when responding to this section. You can access the definitions here.

There are two parts to this section:

1. Crisis - general
2. Suicide

Crisis - general

* 88. If the first aider calls a mental health crisis team, they should find out if they will be attending with the police, and let the person know if this is the case. (Rerate)

- Essential
- Important
- Don't know/depends
- Unimportant
- Should not be included

* 89. If the first aider calls a mental health crisis team or emergency services, they should also let the person's legal guardian know what has happened, if possible. (New)

- Essential
- Important
- Don't know/depends
- Unimportant
- Should not be included

* 90. If the first aider calls a mental health crisis team or emergency services, they should also let the person's family know what has happened, if possible. (New)

- Essential
- Important
- Don't know/depends
- Unimportant
- Should not be included

* 91. If police need to be involved due to illegal behaviours (e.g. aggression, sexually inappropriate behaviour), the first aider should help the person seek an advocate to assist them. (New)

- Essential
- Important
- Don't know/depends
- Unimportant
- Should not be included

* 92. If the crisis mental health team or emergency services refuse to intervene and the first aider feels the person is in need of their assistance, the first aider should strongly advocate for this on the person's behalf. (New)

- Essential
- Important
- Don't know/depends
- Unimportant
- Should not be included

Round 2: Development of guidelines for considerations when providing mental health first aid to a person with an intellectual disability

CRISIS SITUATION cont.

This section contains statements about what the first aider needs to know and do if they are concerned the person is suicidal.

Please rate how important (from ‘essential’ to ‘should not be included’) you think it is that each statement be included in the guidelines.

Please keep our definitions in mind when responding to this section. You can access the definitions here.

Suicide

* 93. If the person has a carer or support person with them, the first aider should share their concerns with them and ask if they are also concerned the person may be at risk of suicide. (Rerate)

- Essential
- Important
- Don't know/depends
- Unimportant
- Should not be included

* 94. If the person is unable to communicate verbally with the first aider and they have a legal guardian, carer or support person with them, the first aider should share their concerns with them and ask if they are also concerned the person may be at risk of suicide. (New)

- Essential
- Important
- Don't know/depends
- Unimportant
- Should not be included

Round 2: Development of guidelines for considerations when providing mental health first aid to a person with an intellectual disability

IF THE PERSON NEEDS TO BE TAKEN TO HOSPITAL

This section contains statements about what the first aider needs to know and do if the person needs to be taken to hospital.

Mental health first aid is the help offered to a person developing a mental health problem, experiencing a worsening of an existing mental health problem, or in a mental health crisis. The first aid is given until appropriate professional help is received or until the crisis resolves.

Therefore, if the person needs to go to hospital, the role of the mental health first aider ceases once the person receives appropriate professional help (e.g. via GP/family doctor, health professional etc). If the first aider is also a carer or support person or disability worker, they may continue to provide ongoing support to the person once they have been seen by a professional. When this occurs, they are no longer acting in their role as a first aider but are providing ongoing support in their usual role. For this reason, this section does not cover ongoing support once the person has received appropriate professional help.

Please rate how important (from ‘essential’ to ‘should not be included’) you think it is that each statement be included in the guidelines.

Please keep our definitions in mind when responding to this section. You can access the definitions here.

* 95. If the person needs to be taken to hospital, the first aider should contact the hospital in advance to make sure they are aware the person has an intellectual disability and if any reasonable adjustments may be needed, with the person's permission. (Rerate)

- Essential
- Important
- Don't know/depends
- Unimportant
- Should not be included

* 96. If the person needs to be taken to hospital, the first aider should find out if the person has any documentation or individual requirements that might be helpful (e.g. hospital passport, communication guidelines, etc) and try to ensure these are made known to hospital staff. (New)

- Essential
- Important
- Don't know/depends
- Unimportant
- Should not be included

Round 2: Development of guidelines for considerations when providing mental health first aid to a person with an intellectual disability

Thank you!

Thank you for sharing your expertise and time with us.

If anything in this survey has caused you distress and you would like to talk with someone about it you can contact the appropriate crisis help line below:

Australia: Lifeline on 13 11 14

Canada: National Suicide prevention Lifeline on 1800 273 TALK (8255)

Denmark: Suicide hotline 70 201 201

Finland: SOS Crisis Centre 010 195 202

France: Suicide Écoute 01 45 39 40 00

The Netherlands: Suicide hotline 0900 0113

New Zealand: Lifeline Aotearoa on 0800 543 354

Republic of Ireland: Samaritans on 116 123

Sweden: Suicide hotline 020 22 00 60

Switzerland: PARSPAS 027 321 21 21

UK: Samaritans on 116 123

USA: National Suicide prevention Lifeline on 1800 273 TALK (8255)

Round 3: Development of guidelines for considerations when providing mental health first aid to a person with an intellectual disability

Information

How this questionnaire was developed
The statements in this questionnaire were derived from the results of the Round 2 survey. This survey includes statements that need to be rerated.

Some of the statements may seem contradictory or controversial; however, they have been included them because they reflect the wide range of people's beliefs about the best ways to provide mental health first aid to a person who has an intellectual disability and is experiencing mental health problems. ***It is important to note that the researchers have not made judgements about the statements in the survey and do not necessarily agree or disagree with them.*** The purpose of the survey is to obtain a consensus view about each of the statements from the expert panel.

Definitions used in this survey

Intellectual disability: is characterised by:
Global deficiencies in intellectual and adaptive functioning, where the person may not reach expected developmental milestones. The deficiencies in the intellectual functions and adaptability begin during childhood or adolescence.

Mental health first aid: is the help offered to a person developing a mental health problem, experiencing a worsening of an existing mental health problem, or in a mental health crisis. The first aid is given until appropriate professional help is received or until the crisis resolves.

The person: a person who has an intellectual disability, who the mental health first aider (first aider) is concerned may be experiencing a mental health problem.

The first aider: a family member, friend, concerned community member or disability worker without specialist mental health qualifications who provides initial help to a person who has an intellectual disability and is experiencing mental health problems.

Carer or support person: refers to people who provide paid or unpaid personal care, support and assistance to a person with an intellectual disability. It can include a spouse, de facto partner, parent, other relative or guardian, or disability support worker.

Disability worker: a person paid to support someone with an intellectual disability. They may or may not be formally trained.

GP/Family doctor: a medical doctor based in the community who treats patients with minor or chronic illnesses and refers those with serious conditions to a specialist or hospital.

Professional/health professional: a broad range of health professionals through which a person may seek help for mental health problems. This could include a mental health professional, GP/family doctor, or another health professional, e.g. allied health professional, hospital emergency staff.

Mental health professional: a health professional who is qualified to treat people who are experiencing mental health problems, e.g. a psychologist, mental health nurse, psychiatrist, or social worker or occupational therapist with specialist mental health training.

Emergency services: services that respond to and deal with emergencies when they occur, e.g. emergency medical services (ambulance) or law enforcement (the police).

Mental health crisis service: services that respond to and provide immediate help during a mental health crisis and are responsible for assessing the care required by the person. Psychiatric nurses, social workers, psychiatrists and psychologists may work for a mental health crisis service.

Mental health crisis/crisis: a situation in which:
- The person may harm themselves, e.g. by attempting suicide, by using substances to become intoxicated, by engaging in non-suicidal self-injury, or as a result of extreme weight loss
- The person experiences extreme distress, e.g. a panic attack, a traumatic event or a severe psychotic state
- The person's behaviour is very disturbing to others, e.g. they become aggressive or lose touch with reality.

Abuse: mistreatment that occurs between people (interpersonal trauma), e.g. emotional, physical or sexual abuse including family violence, child abuse, and elder abuse.

Trauma: an emotional response to a powerful and distressing experience. Immediate trauma responses can include shock and denial. Longer term reactions include unpredictable emotions, flashbacks, strained relationships and physical symptoms like headaches or nausea. Powerful and distressing experiences are usually life threatening or pose a significant threat to a person's physical or psychological wellbeing.

Some common examples events that have the potential to cause trauma include interpersonal violence (including family violence, child abuse, elder abuse, physical or sexual assault, mugging or robbery), accidents (such as traffic or workplace accidents), and witnessing something terrible happen. Mass traumatic events include war, terrorist attacks, mass shootings, and severe weather events (flood, earthquake, hurricane, tsunami, forest and bush fire).

Indirect exposure can also cause trauma, for example witnessing others experience a potentially traumatic event, learning that a potentially traumatic event occurred to someone you know, or repeated or extreme exposure to details of a potentially traumatic event.

Non-suicidal self-injury: self injury that is not intended to result in death. It does not include behaviours that may cause unintentional injury to the person (e.g. stimming behaviours).

Self-injury/self-injurious behaviour: behaviour that has the potential to cause physical injury to oneself and can be for a range of reasons, including to communicate a need such as hunger, to manage distress (NSSI), or as a result of stimming behaviours.

Self-stimulatory behaviour (stimming): the repetition of physical movements, sounds, or repetitive movement of objects common in people with an intellectual disability. Stimming behaviours include hand flapping, rocking, excessive or hard blinking, pacing, head banging, hitting the head, pulling the hair, repeating noises or words, snapping fingers, and spinning objects. While stimming can be an outlet for the person - e.g. to gain/reduce sensory input if the person has sensory sensitivities, some stimming behaviours can cause unintentional injury to the person (e.g. hitting the head or pulling the hair).

'*Challenging behaviour*': Behaviour of such intensity, frequency or duration that the physical safety of the person or others is placed in serious jeopardy, or behaviour which is likely to seriously limit or deny access to the use of ordinary community facilities. It can include a range of behaviours, for example physical aggression towards objects or people, self-injury, sexually inappropriate behaviour, offending behaviour (such as arson or stealing), mannerisms or ritual.

Round 3: Development of guidelines for considerations when providing mental health first aid to a person with an intellectual disability

Instructions

Instructions
There are already guidelines to help people who may be experiencing a range of mental health problems (go to our website to access these guidelines). We do not wish to replicate existing MHFA guidelines. Rather, the aim of this project is to develop guidelines on how to tailor mental health first aid to help a person who has an intellectual disability and is experiencing mental health problems. The guidelines that results from this project will be used in conjunction with the existing mental health first aid guidelines.

Please complete the questionnaire by rating each statement according to how important you believe it is for inclusion in the guidelines for considerations when providing mental health first aid to a who has an intellectual disability and is experiencing mental health problems. Please keep in mind that the guidelines will be used by family members, friends, concerned community members or disability workers without specialist mental health qualifications. The guidelines will not be for mental health professionals who are looking for guidance on working with patients with intellectual disabilities.

The statements need to be rated according to their importance for someone ***WITHOUT clinical background.***

This questionnaire should take approximately 10 minutes to complete. You can complete the survey in two or more sittings. Your answers are saved when you click 'Next' at the bottom of a page. This marks your page and you can begin again at a later date on the next page. **Please be aware that once you have logged on and started responding you must complete the questionnaire on the same computer.**

The next phase of the research involves completing one more short surveys.

For more information
You received a Plain Language Statement when you expressed interest in this project. Please refer to this for more details about this study.

Round 3: Development of guidelines for considerations when providing mental health first aid to a person with an intellectual disability

Information about you

* 1. What is your name? (This allows us to determine who has completed the Round 1 survey and is therefore eligible to participate in the review of the guidelines. Your name will be deleted from your data when the project is complete).

Round 3: Development of guidelines for considerations when providing mental health first aid to a person with an intellectual disability

RECOGNISING AND ACKNOWLEDGING A PERSON MAY BE EXPERIENCING MENTAL HEALTH PROBLEMS

This section contains statements about what the first aider needs to know about recognising and acknowledging that a person with an intellectual disability may be experiencing mental health problems.

Please rate how important (from ‘essential’ to ‘should not be included’) you think it is that each statement be included in the guidelines.

Please keep our definitions in mind when responding to this section. You can access the definitions here.

PLEASE NOTE:
The following three statements apply **only** to situations in which the first aider is a **paid disability worker.**
Please rate how important (from ‘essential’ to ‘should not be included’) you think it is that each statement be included in the guidelines when the first aider is a **paid disability worker.**

* 2. If the person does not consent to the first aider discussing the person's mental health with an appropriate work colleague (e.g. supervisor, manager, case manager), they should seek advice without identifying the person.

- Essential
- Important
- Don't know/depends
- Unimportant
- Should not be included

Round 3: Development of guidelines for considerations when providing mental health first aid to a person with an intellectual disability

RAISING CONCERNS WITH THE PERSON

This section contains statements about what the first aider needs to know about raising their concerns with the person.

Please rate how important (from ‘essential’ to ‘should not be included’) you think it is that each statement be included in the guidelines.

Please keep our definitions in mind when responding to this section. You can access the definitions here.

* 3. The first aider should not assume they know the best way of helping the person and should be guided by any instruction the person, their carer or their legal guardian may give.

- Essential
- Important
- Don't know/depends
- Unimportant
- Should not be included

Round 3: Development of guidelines for considerations when providing mental health first aid to a person with an intellectual disability

COMMUNICATING WITH THE PERSON

Please rate how important (from ‘essential’ to ‘should not be included’) you think it is that each statement be included in the guidelines.

Please keep our definitions in mind when responding to this section. You can access the definitions here.

Comprehension

* 4. If the first aider thinks the person has lost track of the conversation, they should ask the person to share back their understanding of what the first aider has said.

- Essential
- Important
- Don't know/depends
- Unimportant
- Should not be included

Engaging others in the conversation

* 5. The first aider should direct all communication to the person, even if the person has chosen to have someone else present. (New)

- Essential
- Important
- Don't know/depends
- Unimportant
- Should not be included

Round 3: Development of guidelines for considerations when providing mental health first aid to a person with an intellectual disability

RESPECT AND RIGHTS

This section contains statements about what the first aider needs to know about respecting the person and their rights.

Please rate how important (from ‘essential’ to ‘should not be included’) you think it is that each statement be included in the guidelines.

Please keep our definitions in mind when responding to this section. You can access the definitions here.

* 6. If the first aider needs to ask someone else for information, they should get **appropriate consent (i.e. from the person or their legal guardian)** before doing so.

- Essential
- Important
- Don't know/depends
- Unimportant
- Should not be included

Round 3: Development of guidelines for considerations when providing mental health first aid to a person with an intellectual disability

BEING SUPPORTIVE

This section contains statements about what the first aider needs to know about being supportive to a person who may be experiencing mental health problems.

Please rate how important (from ‘essential’ to ‘should not be included’) you think it is that each statement be included in the guidelines.

Please keep our definitions in mind when responding to this section. You can access the definitions here.

* 7. If the first aider observes that the person may need some practical assistance, they should try to facilitate this support, i.e. provide the support themselves or find an appropriate person.

- Essential
- Important
- Don't know/depends
- Unimportant
- Should not be included

Round 3: Development of guidelines for considerations when providing mental health first aid to a person with an intellectual disability

ABUSE

This section contains statements about what the first aider needs to know if they are concerned the person may be experiencing abuse.

Please rate how important (from ‘essential’ to ‘should not be included’) you think it is that each statement be included in the guidelines.

Please keep our definitions in mind when responding to this section. You can access the definitions here.

* 8. If the first aider has reason to believe the person may be experiencing abuse, the first aider should seek advice from an appropriate service as conversations with the person may contaminate evidence that could be critical to future legal proceedings.

- Essential
- Important
- Don't know/depends
- Unimportant
- Should not be included

* 9. If the first aider has reason to believe the person may be experiencing abuse and is in immediate danger **of physical harm**, the first aider should call on their behalf.

- Essential
- Important
- Don't know/depends
- Unimportant
- Should not be included

Round 3: Development of guidelines for considerations when providing mental health first aid to a person with an intellectual disability

OTHER SUPPORTS

This section contains statements about what the first aider needs to know about encouraging other supports.

Please rate how important (from ‘essential’ to ‘should not be included’) you think it is that each statement be included in the guidelines.

Please keep our definitions in mind when responding to this section. You can access the definitions here.

* 10. If the person has used self-help strategies in the past that they found helpful, the first aider should encourage them to ask others to support them in their use.

- Essential
- Important
- Don't know/depends
- Unimportant
- Should not be included

* 11. If the person needs additional support, but family and friends are under stress or 'burnt out', the first aider should assist the person to find this support.

- Essential
- Important
- Don't know/depends
- Unimportant
- Should not be included

Round 3: Development of guidelines for considerations when providing mental health first aid to a person with an intellectual disability

DIFFICULTIES THE FIRST AIDER MAY ENCOUNTER

This section contains statements about what a first aider should know and do regarding difficulties they may encounter.

Please rate how important (from ‘essential’ to ‘should not be included’) you think it is that each statement be included in the guidelines.

Please keep our definitions in mind when responding to this section. You can access the definitions here.

* 12. If the person is exhibiting behaviours that the first aider finds challenging, the first aider should try to redirect the person away from those behaviours, e.g. "I can see you're really upset, do you want to go for a walk?"

- Essential
- Important
- Don't know/depends
- Unimportant
- Should not be included

* 13. If the person is exhibiting behaviours that the first aider finds challenging, the first aider should try to make changes in the environment (e.g. turning off the TV or asking people to be quiet) as the person may be responding to overstimulation.

- Essential
- Important
- Don't know/depends
- Unimportant
- Should not be included

Round 3: Development of guidelines for considerations when providing mental health first aid to a person with an intellectual disability

PROFESSIONAL HELP

This section contains statements about what the first aider needs to know and do when encouraging the person to seek professional help.

Please rate how important (from ‘essential’ to ‘should not be included’) you think it is that each statement be included in the guidelines.

Please keep our definitions in mind when responding to this section. You can access the definitions here.

* 14. If the person has a legal guardian, the first aider should involve them with regards to seeking professional help. (New)

- Essential
- Important
- Don't know/depends
- Unimportant
- Should not be included

Round 3: Development of guidelines for considerations when providing mental health first aid to a person with an intellectual disability

CRISIS SITUATION

This section contains statements about what the first aider needs to know and do if the person is in crisis.

Please rate how important (from ‘essential’ to ‘should not be included’) you think it is that each statement be included in the guidelines.

Please keep our definitions in mind when responding to this section. You can access the definitions here.

* 15. If police need to be involved due to illegal behaviours (e.g. aggression, sexually inappropriate behaviour), the first aider should help the person seek an advocate to assist them.

- Essential
- Important
- Don't know/depends
- Unimportant
- Should not be included

Round 3: Development of guidelines for considerations when providing mental health first aid to a person with an intellectual disability

Thank you!

Thank you for sharing your expertise and time with us.

If anything in this survey has caused you distress and you would like to talk with someone about it you can contact the appropriate crisis help line below:

Australia: Lifeline on 13 11 14

Canada: National Suicide prevention Lifeline on 1800 273 TALK (8255)

Denmark: Suicide hotline 70 201 201

Finland: SOS Crisis Centre 010 195 202

France: Suicide Écoute 01 45 39 40 00

The Netherlands: Suicide hotline 0900 0113

New Zealand: Lifeline Aotearoa on 0800 543 354

Republic of Ireland: Samaritans on 116 123

Sweden: Suicide hotline 020 22 00 60

Switzerland: PARSPAS 027 321 21 21

UK: Samaritans on 116 123

USA: National Suicide prevention Lifeline on 1800 273 TALK (8255)
